# Regulation of Brown Fat Adipogenesis by Protein Tyrosine Phosphatase 1B

**DOI:** 10.1371/journal.pone.0016446

**Published:** 2011-01-31

**Authors:** Kosuke Matsuo, Ahmed Bettaieb, Naoto Nagata, Izumi Matsuo, Heike Keilhack, Fawaz G. Haj

**Affiliations:** 1 Nutrition Department, University of California Davis, Davis, California, United States of America; 2 Beth Israel Deaconess Medical Center, Harvard Medical School, Boston, Massachusetts, United States of America; Cedars-Sinai Medical Center, United States of America

## Abstract

**Background:**

Protein-tyrosine phosphatase 1B (PTP1B) is a physiological regulator of insulin signaling and energy balance, but its role in brown fat adipogenesis requires additional investigation.

**Methodology/Principal Findings:**

To precisely determine the role of PTP1B in adipogenesis, we established preadipocyte cell lines from wild type and PTP1B knockout (KO) mice. In addition, we reconstituted KO cells with wild type, substrate-trapping (D/A) and sumoylation-resistant (K/R) PTP1B mutants, then characterized differentiation and signaling in these cells. KO, D/A- and WT-reconstituted cells fully differentiated into mature adipocytes with KO and D/A cells exhibiting a trend for enhanced differentiation. In contrast, K/R cells exhibited marked attenuation in differentiation and lipid accumulation compared with WT cells. Expression of adipogenic markers PPARγ, C/EBPα, C/EBPδ, and PGC1α mirrored the differentiation pattern. In addition, the differentiation deficit in K/R cells could be reversed completely by the PPARγ activator troglitazone. PTP1B deficiency enhanced insulin receptor (IR) and insulin receptor substrate 1 (IRS1) tyrosyl phosphorylation, while K/R cells exhibited attenuated insulin-induced IR and IRS1 phosphorylation and glucose uptake compared with WT cells. In addition, substrate-trapping studies revealed that IRS1 is a substrate for PTP1B in brown adipocytes. Moreover, KO, D/A and K/R cells exhibited elevated AMPK and ACC phosphorylation compared with WT cells.

**Conclusions:**

These data indicate that PTP1B is a modulator of brown fat adipogenesis and suggest that adipocyte differentiation requires regulated expression of PTP1B.

## Introduction

The obesity epidemic has focused attention on adipose tissue and adipocyte development (adipogenesis). Adipose tissue is an important metabolic organ that integrates a wide array of homeostatic processes and is crucial for whole-body insulin sensitivity and energy metabolism [1]. White adipose tissue (WAT) is the primary site for triglyceride storage and fatty acid release in response to various energy requirements; whereas brown adipose tissue (BAT) generates heat via mitochondrial uncoupling of lipid oxidation [2]. Brown adipose is a key thermogenic tissue with a well-established role in the defense against cold in a process termed nonshivering thermogenesis [3]. In addition, BAT is recognized for its anti-obesity properties with the increase in brown adipose amount and/or function promoting a healthy phenotype. Specifically, mice with higher amounts of BAT gain less weight, are more insulin sensitive, and are protected from diabetes [4], [5], [6], [7]. Interest in the regulation and development of BAT gained traction in recent years with the realization that adult humans have distinct brown adipose tissue depots and that the activity of BAT varies depending on adiposity, temperature, gender and age [8], [9], [10], [11].

Adipocyte differentiation is a complex process that requires integration of a multitude of stimuli including nutrients and hormones [12], [13], [14], [15]. Despite differences in physiological function and developmental origins of WAT and BAT, both share similar canonical transcriptional cascades that control fat differentiation [16]. Previous detailed studies of WAT differentiation identified peroxisome proliferator-activated receptor gamma (PPARγ) and CCAAT/enhancer-binding proteins (C/EBPs) as critical transcription factors regulating differentiation (reviewed in [17]). PPARγ is also necessary for brown fat cell development but not sufficient to drive mesenchymal cells into a brown fat cell fate. Recently, bone morphogenic protein 7 (BMP7) was identified as a regulator of brown fat cell differentiation program [18]. In addition, insulin and insulin-like growth factor 1 (IGF1) play important roles in brown adipocyte differentiation [19]. Brown preadipocytes derived from insulin receptor (IR) and insulin receptor substrates 1–4 (IRSs) knockout (KO) mice highlight the relevance of upstream components in insulin signaling in BAT differentiation [20], [21], [22], [23].

Tyrosyl phosphorylation is a major regulator of insulin signaling and is tightly controlled by the opposing actions of protein-tyrosine kinases (PTKs) and protein-tyrosine phosphatases (PTPs) [24], [25]. Protein-tyrosine phosphatase 1B (PTP1B) is an abundant, widely expressed non-receptor tyrosine-specific phosphatase that is localized on the cytoplasmic face of the endoplasmic reticulum (ER) [26], [27], [28]. Whole-body PTP1B deficient mice are hypersensitive to insulin, lean and resistant to high fat diet-induced obesity [29], [30]. The leanness is caused by increased energy expenditure that is mediated, at least in part, by neuronal PTP1B since neuron-specific PTP1B KO mice exhibit reduced body weight and increased energy expenditure [31]. In contrast, muscle- and liver-specific PTP1B deletion leads to improved insulin sensitivity without alterations in body weight [32], [33]. However, the role of PTP1B in adipose tissue, specifically BAT is less clearly defined. Of note, whole-body PTP1B deficient mice exhibit increased AMP-activated protein kinase (AMPK) activity and mitochondrial content in BAT [34]. In addition, it was recently reported that PTP1B deficiency has a beneficial effect on brown adipocyte differentiation and protection against apoptosis [35].

In the current study we investigated the role of PTP1B in brown adipocyte differentiation and signaling. We utilized preadipocytes from WT and PTP1B-deficient mice and reconstituted cells to reveal that PTP1B modulates brown fat adipogenesis. In addition, we demonstrated a role for PTP1B in regulating insulin and AMPK signaling in brown adipocytes.

## Results

### Generation and characterization of PTP1B-deficient and reconstituted brown adipocytes

**Figure 1:**
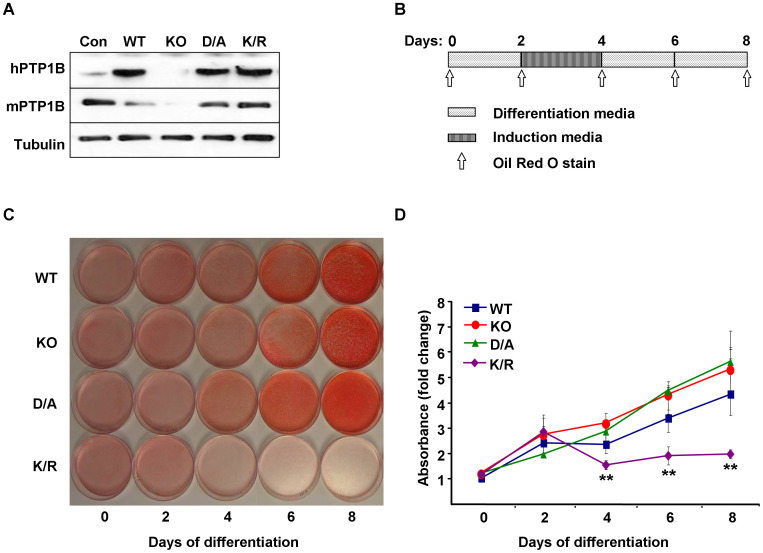
PTP1B regulates brown adipocyte differentiation. **A**) Immunoblots of human (h) and mouse (m) PTP1B expression in immortalized brown adipocytes from wild type mice (Con), PTP1B knockout (KO) mice, and KO cells reconstituted with hPTP1B (WT), substrate-trapping (D/A), and sumoylation-resistant (K/R) hPTP1B mutants. Blots were probed with anti-Tubulin antibodies as a control for loading. **B**) Schematic depicting timeline of cell differentiation and oil red O staining. **C**) Brown adipose precursor cells were grown to confluence, then differentiation was induced as described in Methods. At various stages of differentiation, cells were fixed and stained with oil red O, then the dye was extracted and its absorbance (520 nm) quantitated (**D**). Graph represents data from nine independent experiments, and data are expressed as mean ± SEM. (**) indicates significant difference between K/R and WT, KO and D/A cells.

To investigate the role of PTP1B in brown adipocyte differentiation, we generated immortalized brown preadipocytes from wild type (Con) and whole-body PTP1B KO mice as described in Methods. To determine whether alterations in KO cells were directly caused by PTP1B deficiency, we generated isogenic cells by reconstituting KO cells with human (h) PTP1B (WT) as described in Methods. Of note, hPTP1B shares a high degree of homology to mouse (m) PTP1B, and we have previously demonstrated that hPTP1B can rescue the effects of mPTP1B deletion in mouse embryonic fibroblasts in response to growth factors stimulation [36]. In addition, KO cells were reconstituted with substrate-trapping hPTP1B D181A (D/A) mutant that retains substrate binding but is catalytically impaired [37], and sumoylation-resistant mutant hPTP1B K73, 335, 347, 389R (K/R) [38]. PTP1B is progressively sumoylated after insulin stimulation leading to inhibition of its catalytic activity and suppression of its ability to downregulate the IR [38]. Immunoblot analysis of cell lysates revealed that hPTP1B was expressed in all reconstituted cells (WT, D/A, and K/R) while mouse (m) PTP1B was expressed in Con cells and absent in KO cells (Fig. 1A). Given the cross reactivity of mouse and human PTP1B antibodies [39], we estimated that hPTP1B expression in reconstituted cells was roughly comparable to mPTP1B in wild type cells (Con). Differentiation of KO and reconstituted cells into brown adipocytes was performed as described in Methods and outlined in Fig. 1B. Cells were stained using the fat-specific dye oil red O to monitor lipid accumulation at various days of differentiation (Fig. 1B, C). Differentiation was quantitated by extracting oil red O from stained cells and determining absorbance (from 9 independent experiments) (Fig. 1D). WT cells accumulated fat droplets and exhibited a fully differentiated phenotype with >90% of the cells containing multilocular fat droplets at day 8 (Fig. 1C). KO and D/A cells exhibited a trend for enhanced differentiation compared with WT cells, but did not reach statistical significance (Fig. 1C, D). In contrast, K/R cells treated with the same protocol failed to differentiate with only a small percentage of cells able to accumulate fat. Together, our findings indicate that differentiation of brown adipocytes requires a regulated expression of PTP1B and that its sumolyation-resistant mutant dramatically inhibits differentiation.

### Patterns of differentiation correlate with expression of adipogenic markers

**Figure 2:**
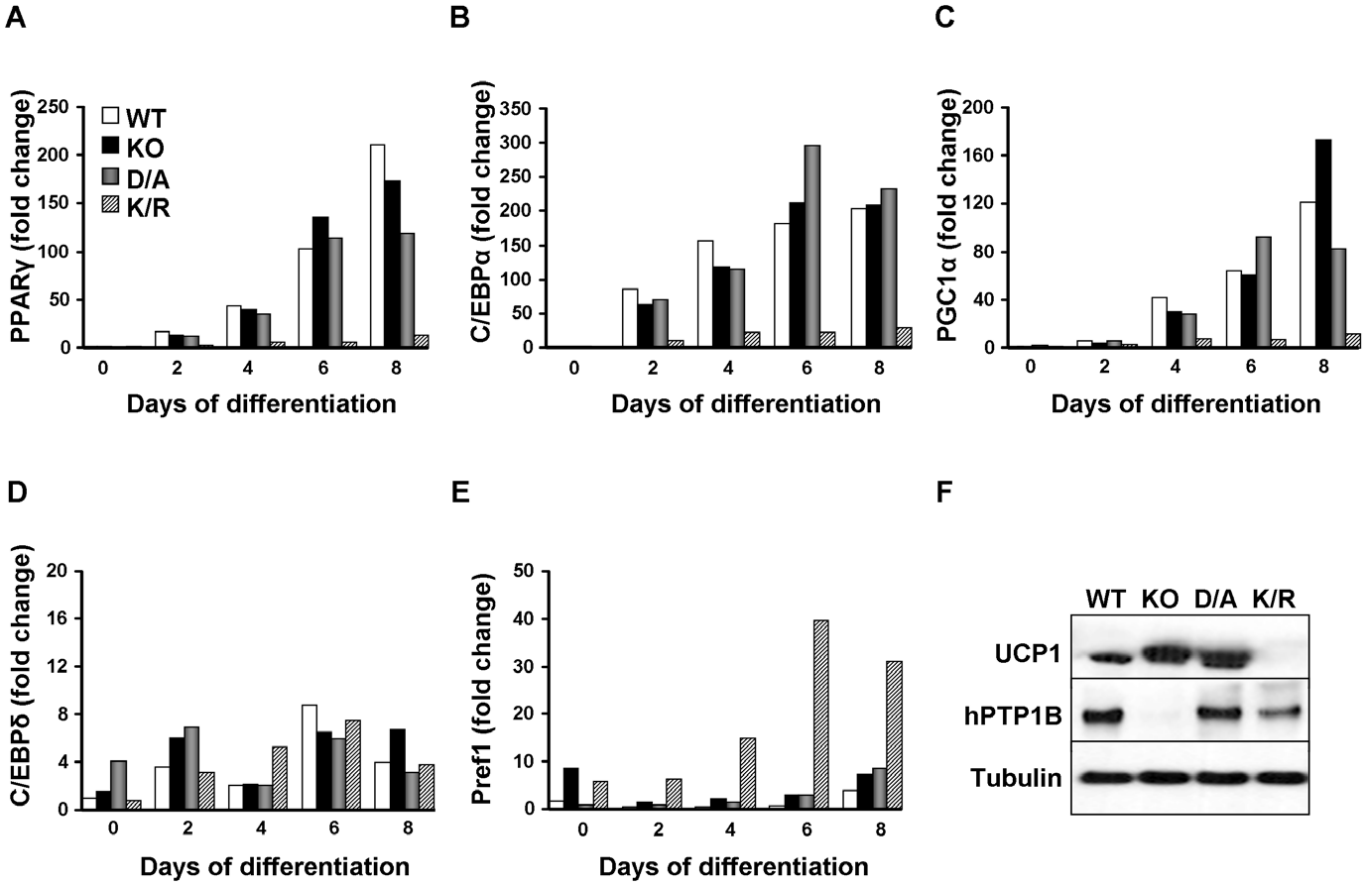
Expression of differentiation markers in PTP1B KO and reconstituted adipocytes. mRNA expression of PPARγ (**A**), C/EBPα (**B**), PGC1α (C), C/EBPδ (**D**) and Pref1 (**E**) at different days of differentiation, measured by quantitative real-time PCR and normalized against GAPDH. Results are representative of two independent experiments and data are expressed as mean ± SEM. (**F**) Immunoblots of UCP1 expression in differentiated cells. Blots were probed for hPTP1B and with anti-Tubulin antibodies as a control for loading.

To further investigate the role of PTP1B in brown adipose differentiation, we determined the expression of adipogenic markers PPARγ, C/EBPα, C/EBPδ, PPARγ coactivator 1α (PGC1α) and preadipocyte factor 1 (Pref1) mRNAs in KO and reconstituted cells during differentiation. Consistent with previous reports [23], [40], PPARγ exhibited a progressive increase in expression during differentiation in WT cells (Fig. 2A). Notably, KO and D/A cells revealed a comparable pattern of PPARγ expression, while K/R cells exhibited blunted expression throughout differentiation. Similarly, C/EBPα exhibited comparable expression pattern to that of PPARγ. Transcripts of C/EBPα peaked on day 4–6 in WT, KO and D/A cells, while K/R cells exhibited blunted expression (Fig. 2B). In addition, PGC1α mRNA expression pattern was comparable to that of PPARγ increasing progressively during differentiation in WT, KO and D/A cells while K/R cells exhibited blunted expression (Fig. 2C). C/EBPδ levels were generally low in all cells and no apparent trend was observed (Fig. 2D). On the other hand, expression of Pref1, an inhibitor of adipocyte differentiation (reviewed in [41]), was elevated in K/R cells compared with KO, WT and D/A cells (Fig. 2E). Finally, protein expression of uncoupling protein 1 (UCP1), a marker of brown adipocyte differentiation, was elevated in differentiated KO and D/A cells compared with WT and was not detectable in K/R cells. This is in line with *in vivo* studies that report increased UCP1 expression (by immuno-blotting and immuno-histochemistry) in BAT of PTP1B KO mice compared with wild type mice [34]. Therefore, K/R cells exhibited attenuated differentiation, as indicated by lipid accumulation, mRNA and protein expression.

### Tyrosyl phosphorylation and differentiation of PTP1B-deficient and reconstituted adipocytes

**Figure 3:**
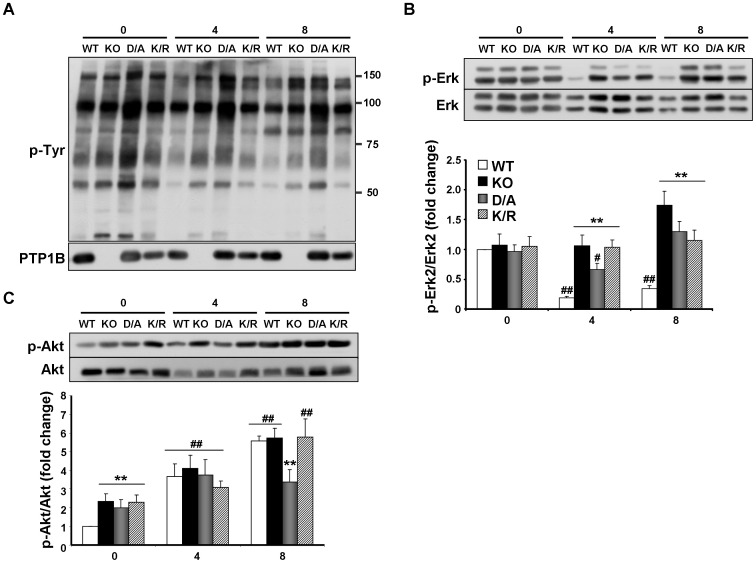
Overall tyrosyl phosphorylation, Erk and Akt phosphorylation during differentiation. **A**) Overall tyrosyl phosphorylation in WT, KO, D/A and K/R cells during differentiation. Total lysates were prepared from cells on days 0, 4 and 8 of differentiation. Blots were probed using anti-phosphotyrosine and anti-hPTP1B antibodies. Molecular weight markers are indicated on the right of the blot. Total lysates from cells at days 0, 4 and 8 of differentiation were immunoblotted for p-Erk (**B**) and p-Akt (**C**) and the corresponding proteins. (*) indicates statistically significant difference between KO, D/A or K/R and WT under the same treatment condition, while (^#^) indicates statistically significant difference between WT, KO, D/A or K/R and their corresponding basal (day 0).

Overall tyrosyl phosphorylation inversely correlates with adipocyte differentiation [23]; we reasoned that PTP1B deletion and/or reconstitution will likely alter tyrosyl phosphorylation and modulate differentiation. Overall tyrosyl phosphorylation was determined in lysates of KO and reconstituted adipocytes at various stages of differentiation (Fig. 3A). As differentiation progressed, we detected a trend for mild decrease in tyrosyl phosphorylation; however the overall pattern and levels were comparable in KO, WT and K/R cells (Fig. 3A). In line with this, we observed a trend of increased PTP1B expression in WT cells during differentiation, but it did not reach statistical significance (data not shown). Please note that enhanced tyrosyl phosphorylation in D/A cells likely reflects “trapped” PTP1B substrates that are protected against dephosphorylation. Previous studies indicate that Erk phosphorylation decreases during the progression of differentiation [22], [23]. Indeed, Erk phosphorylation declined in WT cells during differentiation, while KO, D/A and K/R cells failed to attenuate Erk phoshorylation (Fig. 3B). Finally, PI3K and its downstream effector, Akt play an important role in brown adipocyte differentiation [20]. Akt (Ser473) phopshorylation, adjusted to Akt expression, increased as differentiation proceeded in all cells (Fig. 3C). In addition, Akt phosphorylation was higher in KO, D/A and K/R cells compared with WT at day 0, but these differences were not sustained at later stages of differentiation (Fig. 3C). Thus, while some differences in Erk and Akt phosphorylation were observed between cells during differentiation, these are unlikely to account, on their own, for the altered differentiation pattern in these cells.

### Regulation of insulin signaling in brown adipocytes by PTP1B

**Figure 4:**
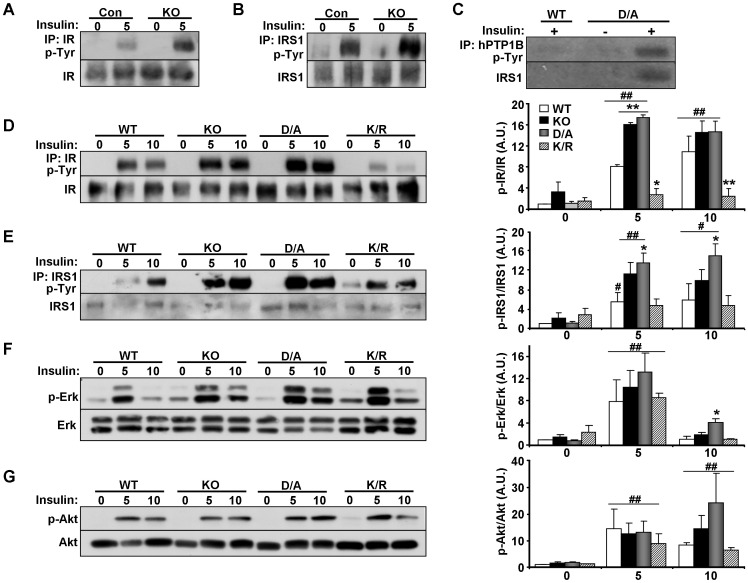
Altered insulin signaling in PTP1B KO and reconstituted adipocytes. Brown adipocyte cell lines from WT (Con) and PTP1B KO (KO) mice were differentiated then serum starved O/N and stimulated with insulin (100 nM) for 5 minutes. **A**) Lysates were immunoprecipitated using IR antibodies, immunoblotted with anti-phosphotyrosine antibodies, and then stripped and reprobed for IR to control for loading. **B**) IRS1 was immunoprecipitated from adipocyte lysates, immunoblotted with anti-phosphotyrosine antibodies, and then stripped and reprobed for IRS1 to control for loading. **C**) Lysates from cells reconstituted with WT and D/A were immunoprecipitated using hPTP1B antibodies (FG6) followed by immunoblotting with anti-phosphotyrosine and anti-IRS1 antibodies. (**D–G**) Isogenic KO and reconstituted differentiated cells were serum starved O/N then stimulated with insulin for 5 or 10 minutes. **D**) Lysates were immunoprecipitated using IR antibodies, immunoblotted with anti-phosphotyrosine antibodies, and then stripped and reprobed for IR to control for loading. **E**) IRS1 was immunoprecipitated from adipocyte lysates, immunoblotted with anti-phosphotyrosine antibodies, and then stripped and reprobed for IRS1 to control for loading. Cell lysates were immunoblotted for p-Erk (**F**) p-Akt (**G**) and the corresponding total proteins. Bar graphs are data from three independent experiments and are presented as means ± SEM. (*) indicates statistically significant difference between KO, D/A or K/R and WT under the same treatment condition, while (^#^) indicates statistically significant difference between WT, KO, D/A or K/R and their corresponding basal (no insulin).

Insulin signaling is an important regulator of brown fat adipogenesis with upstream components, IR [22] and IRS1/3 [20], [23], [42] playing crucial roles. PTP1B attenuates insulin signaling by dephosphorylating IR [43], [44] and possibly IRS1 [45], [46]. Initially, we determined alterations in insulin signaling in differentiated brown adipocytes derived from wild type (Con) and KO mice. Cells were starved overnight then stimulated with insulin for 5 minutes and tyrosyl phosphorylation of IR and IRS1 was determined in immunoprecipitates. Insulin-stimulated tyrosyl phosphorylation of IR and IRS1 was significantly enhanced in KO cells compared with controls (Fig. 4A, B). Enhanced insulin-induced IRS1 tyrosyl phosphorylation in KO cells could be primary indicating that IRS1 is a substrate of PTP1B, or secondary due to increased IR phosphorylation in KO cells. To determine if PTP1B directly interacts with IRS1 in differentiated brown adipocytes, we performed substrate-trapping experiments as previously described [37]. hPTP1B was immunoprecipitated (using FG6 antibodies) from lysates of WT and D/A cells then blotted using anti-phosphotyrosine antibodies (Fig. 4C). Notably, a hyper-phosphorylated band, whose size corresponds to IRS1 was detected in PTP1B immunoprecipitates of insulin-stimulated D/A cells. Indeed, reprobing with IRS1 antibodies identified the protein as IRS1 demonstrating that it is a direct target of PTP1B in brown adipocytes.

Next, we evaluated alterations in insulin signaling in isogenic KO and reconstituted differentiated adipocytes. Insulin-stimulated IR tyrosyl phosphorylation, normalized to its expression, was elevated in KO and D/A cells compared with WT cells (Fig. 4D). By contrast, IR phosphorylation was significantly decreased in K/R cells consistent with the inability of insulin to suppress activity of PTP1B K/R [38]. Similarly, insulin-stimulated IRS1 tyrosyl phosphorylation, normalized to its expression, was elevated in KO and D/A cells compared with WT cells, and K/R cells exhibited comparable IRS1 phosphorylation to WT cells (Fig. 4E). Downstream insulin signaling was assessed by determining basal and insulin-stimulated Erk and Akt phosphorylation in differentiated adipocytes. Erk phosphorylation was significantly induced 5 mins after insulin stimulation compared with basal, but no major differences were observed between cells (Fig. 4F). Similarly, Akt phosphorylation was significantly induced after insulin stimulation compared with basal, but no major differences were observed between cells (Fig. 4G). Together, our findings indicate that PTP1B is a regulator of IR signaling and that IRS1 is a direct PTP1B substrate in brown adipocytes.

### Regulation of AMPK signaling in brown adipocytes by PTP1B

**Figure 5:**
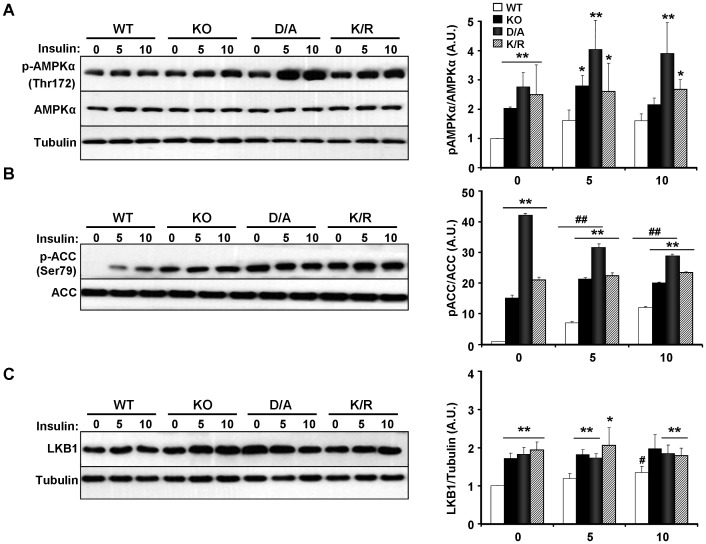
Altered AMPK signaling in PTP1B KO and reconstituted adipocytes. **A**) Immunoblotting of AMPKα subunit phosphorylation (Thr172) in differentiated KO and reconstituted brown adipocytes under starved and insulin-stimulated conditions. Phosphorylation of ACC (Ser79) (**B**), and LKB1 expression (**C**) in differentiated WT, KO, D/A and K/R cells at basal and insulin-stimulated conditions. Bar graphs are data from three independent experiments and are presented as means ± SEM. (*) indicates statistically significant difference between KO, D/A or K/R and WT under the same treatment condition, while (^#^) indicates statistically significant difference between WT, KO, D/A or K/R and their corresponding basal (no insulin). Editors' note: The Fig. 5A Tubulin panel was originally published as a STAT3 panel in [63], and is reused here under the terms of that article's CC-BY-4.0 DEED license. The editors recommend that the Fig. 5A results are interpreted with caution.

AMPK is a fuel sensing enzyme complex and a regulator of brown adipose differentiation [47]. BAT from whole-body PTP1B KO mice exhibit increased α1 and α2 AMPK activation, as well as increased phosphorylation of α1 subunit at Thr172 compared with controls [34]. In line with these observations, phosphorylation of AMPKα (Thr172), adjusted to AMPKα expression was elevated in KO and D/A cells compared with controls (Fig. 5A). In addition, K/R cells exhibited elevated AMPKα phosphorylation compared with controls. AMPK activation leads to phosphorylation of its downstream target acetyl-CoA carboxylase (ACC) at Ser79 and ACC inhibition [48], [49]. Consistent with AMPKα phosphorylation, ACC phosphorylation (Ser79) was elevated in KO, D/A and K/R cells compared with controls (Fig. 5B). LKB1 is one of the upstream kinases that activate AMPKα by phosphorylating Thr172 [50], [51]. LKB1 protein levels were elevated in KO, D/A and K/R cells compared with controls (Fig. 5C) and likely to contribute to increased AMPK phophorylation in these cells.

### Insulin-stimulated glucose uptake is impaired in PTP1B K/R adipocytes

**Figure 6:**
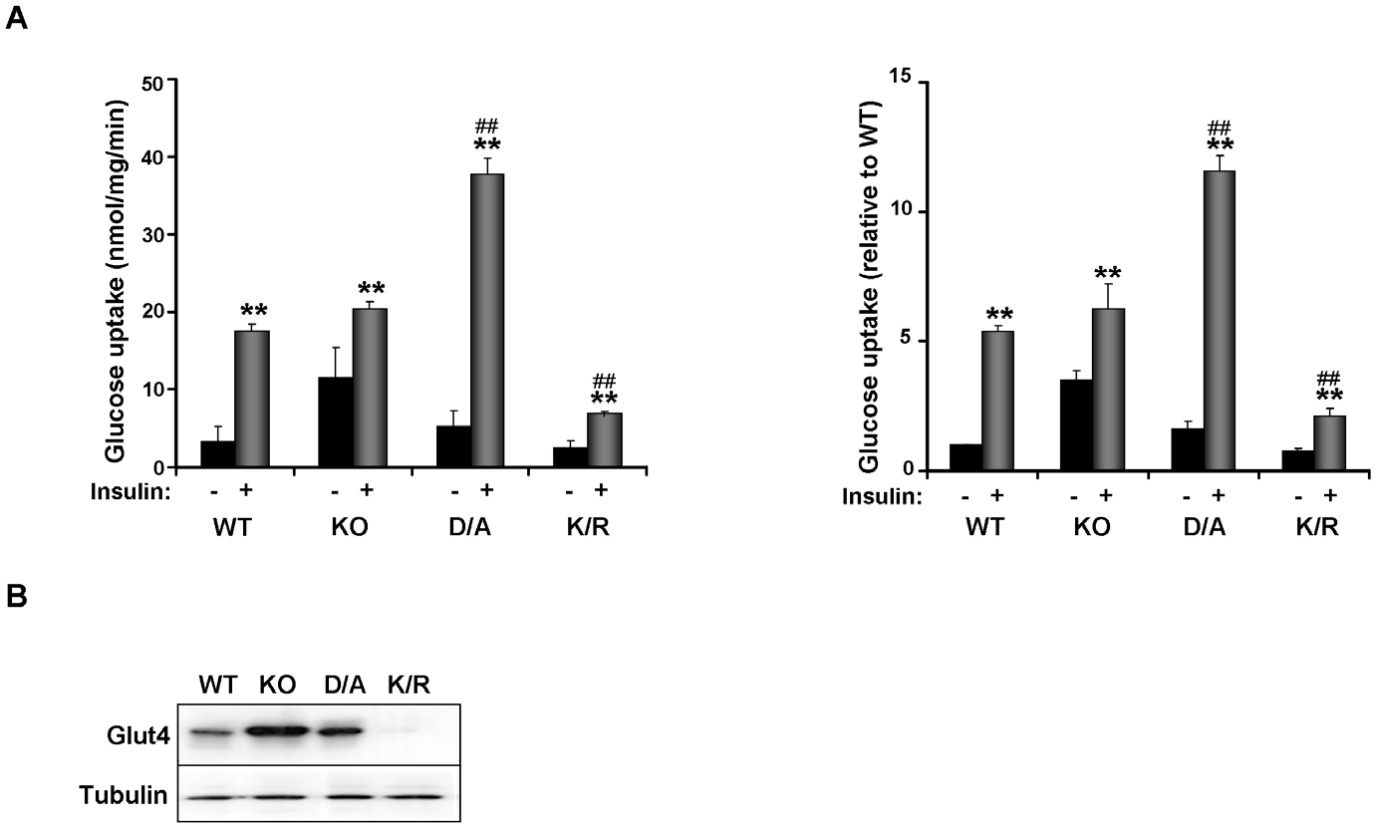
Insulin-induced glucose uptake is impaired in K/R adipocytes. **A**) Differentiated KO and reconstituted cells were serum starved O/N then stimulated with insulin (100 nM) for 30 minutes followed by addition of 2-deoxy-[^3^H] glucose for 3 minutes. Data are expressed as nmol of glucose taken up/mg of cell protein/min (left panel) and as percentage of basal uptake in WT cells (right panel). Bar graphs are data from three independent experiments and data are presented as means ± SEM. **B**) Immunoblots of Glut4 expression in differentiated cells. Blots were probed with anti-Tubulin antibodies as a control for loading. (*) indicates statistically significant difference between basal and insulin-stimulated conditions in each cell type, while (^#^) indicates statistically significant difference between insulin-stimulated glucose uptake in KO, D/A and K/R cells compared with WT.

Basal and insulin-stimulated 2-deoxy-glucose uptake was determined in differentiated KO and reconstituted cells as described in Methods. There was no significant difference in basal glucose transport between cells, although KO cells exhibited a trend for increased glucose uptake compared with WT, but that did not reach statistical significance (Fig. 6A). After insulin stimulation, WT cells exhibited a significant increase in glucose uptake (∼5 fold) in line with published observations [20], [21]. A comparable increase in insulin-stimulated glucose uptake was observed in KO cells. In addition, insulin-induced glucose uptake was significantly enhanced in D/A cells and attenuated in K/R cells compared with WT (Fig. 6A). Decreased insulin-induced glucose uptake in K/R cells was paralleled by attenuated Glut4 expression in these cells (Fig. 6B). On the other hand, elevated glucose uptake in D/A cells was not mirrored by comparable increase in Glut4 expression in total lysates. This suggests that other factor(s), including but not limited to Glut4 translocation, could be altered in these cells. At any rate, our findings demonstrated impaired insulin-stimulated glucose uptake in K/R brown adipocytes.

### Troglitazone treatment increases differentiation capacity of K/R cells

**Figure 7:**
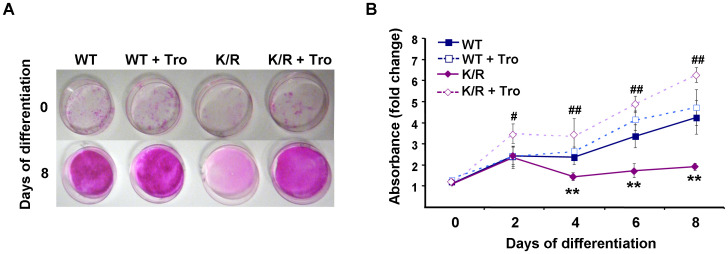
Differentiation capacity of K/R cells is improved by troglitazone treatment. WT and K/R cells were induced to differentiate in the absence or presence of 25 µM troglitazone. **A**) At differentiation days 0 and 8 cells were stained with oil red O and differentiation quantitated (**B**). Graph represents data from three independent experiments, and data are expressed as mean ± SEM. (*) indicates statistically significant difference between untreated K/R and WT cells, and (^#^) indicates statistically significant difference between untreated K/R and troglitazone-treated K/R cells.

K/R cells failed to accumulate the adipogenic marker PPARγ and given its role in brown and white adipose differentiation [17], we determined the effects of PPARγ activation on the differentiation capacity of K/R cells. WT and K/R cells were treated with the PPARγ agonist troglitazone and differentiation was monitored. Addition of troglitazone to WT cells did not significantly alter their differentiation profile probably due to the natural differentiation potential of these cells (Fig. 7A, B). On the other hand, PPARγ activation significantly improved the ability of K/R cells to differentiate as measured by oil red O staining (Fig. 7A, B). Thus, troglitazone treatment is sufficient to override the differentiation deficit in K/R cells.

## Discussion

Adipocyte differentiation requires integration of a multitude of stimuli and coordinated regulation of cellular responses [12], [13], [14], [15]. Despite recent confluence of discoveries on brown fat adipogenesis, molecular regulators of this complex mechanism are not fully identified. PTP1B is an established physiological regulator of systemic insulin sensitivity and energy balance, but its role in brown fat adipogenesis warrants additional investigation. In this study we utilized immortalized brown preadipocytes from wild type and PTP1B KO mice and reconstituted KO preadipocytes to address the role of PTP1B in adipogenesis. These cells provide a useful platform since preadipocytes can differentiate into mature brown adipocytes with accumulation of multilocular fat droplets and expression of adipogenic and differentiation markers [22], [42]. In addition, these cells are an established model for dissecting the contribution of components in insulin signaling to brown fat adipogenesis and glucose uptake [20], [23], [42]. Moreover, PTP1B-reconstituted cells help identify if observed alterations in KO cells are directly caused by PTP1B deletion.

Using standard differentiation protocols, KO and D/A preadipocytes exhibited a trend (that did not reach statistical significance) for increased differentiation and accumulation of fat droplets compared with WT cells. These findings are in line with those of Miranda *et al.* who report beneficial effects of PTP1B deficiency on brown fat adipogenesis [35]. Conversely, differentiation of preadipocytes expressing a sumoylation-resistant PTP1B mutant (K/R) was dramatically reduced compared with controls. The underlying reason(s) for attenuated K/R preadipocyte differentiation is not clear. One scenario involves attenuated insulin signaling in these cells (see next paragraph). Another possibility involves alteration of signaling in distinct cellular compartment(s). The bulk of sumoylated PTP1B localizes to the perinuclear region [38], and since K/R is resistant to insulin-induced downregulation, then presumably K/R cells manifest increased and/or prolonged PTP1B activation at this region. Therefore, K/R could modulate insulin (and potentially other) signaling amplitude and/or duration to attenuate differentiation. In addition, we cannot rule out that K/R regulates a distinct set of cellular substrate(s), and this warrants additional investigation. At any rate, treatment of K/R cells with the PPARγ agonist troglitazone fully recovers the differentiation blockade in these cells. This could be due to direct activation of PPARγ, and/or indirectly caused by the insulin-sensitizing effects of thiazolidinedione. Of note, troglitazone treatment of IR-deficient brown preadipocytes slightly improves their differentiation profile [22], and in IRS1-deficient preadipocytes reverses some of their differentiation deficits (Glut4 expression) without affecting others (fat accumulation) [20]. Although the exact mechanism of impaired differentiation of K/R cells requires additional investigation, our results demonstrate that PTP1B regulates brown fat adipogenesis.

**Figure 8:**
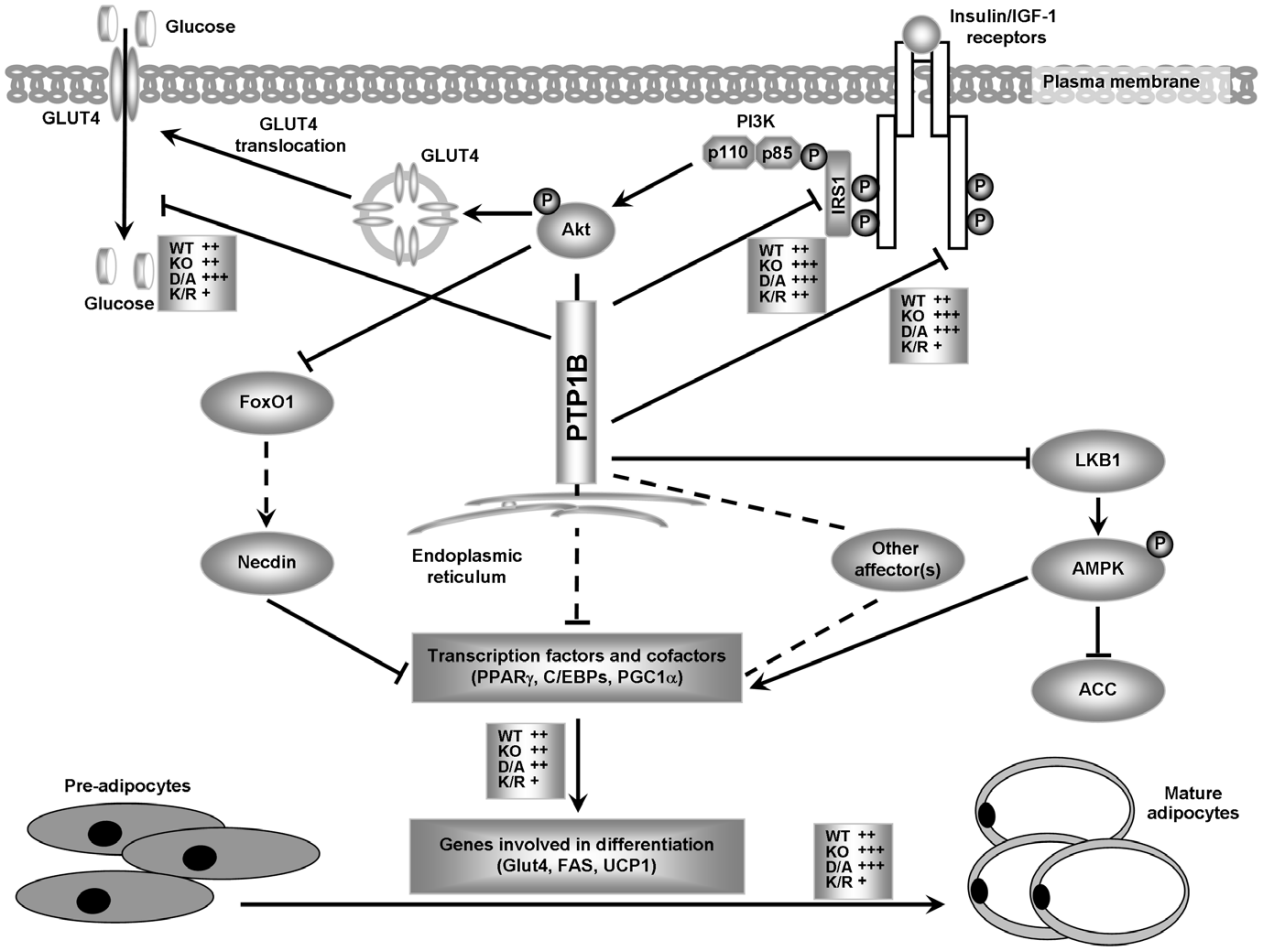
Proposed model of regulation of brown fat adipogenesis by PTP1B. PTP1B can regulate adipogenesis through insulin-dependent and insulin-independent pathways. Activation of cell surface receptors of insulin or IGF1 leads to phosphorylation of IRS proteins, activation of PI3K and MAPK pathway, and glucose uptake. Alterations in downstream signaling (summarized in Tseng *et al.* [23], [40]) lead to the initiation of transcriptional cascades that involve PPARγ, C/EBPs, and PGC1α and cause changes that are required for differentiation of brown adipocytes (expression of Glut4 and UCP1). PTP1B dephosphorylates IR and IRS1 in differentiated brown adipocytes and modulates insulin-stimulated glucose uptake. In addition, PTP1B regulates LKB1/AMPK signaling pathway in differentiated brown adipocytes. Conceivably, PTP1B also could modulate other affector(s) of cellular signaling to regulate transcription factors and cofactor to influence differentiation. Effects of KO, D/A and K/R on adipose differentiation, IR and IRS1 tyrosyl phosphorylation, glucose uptake, and LKB1/AMPK signaling are summarized. Alterations in KO, D/A and K/R are indicated with the symbol (+) and compared with WT (set at ++). Arrows indicate activation and T-shaped lines indicate repression. A dashed line indicates that the connection has not been completely established.

PTP1B can regulate brown fat adipogenesis through insulin-dependent and insulin-independent signaling pathways (please see schematic in Fig. 8). Numerous studies establish insulin signaling as a critical regulator of brown fat adipogenesis [20], [21], [22], [23], [42]. Brown preadipocyte cell lines from IR KO mice exhibit dramatically impaired differentiation [22]. Similarly, preadipocytes from IRS1 KO mice exhibit a marked decrease in differentiation and lipid accumulation [20], [23], [42]. In addition, expression of adipogenic makers and transcription factors (such as PPARγ, C/EBPα, PGC1α, Glut4, and fatty acid synthase) is attenuated in preadipocytes from IR and IRS1 KO mice [20], [22], [23]. Our studies clearly established PTP1B as a regulator of IR and IRS1 signaling in differentiated brown adipocytes and demonstrated that IRS1 is a substrate of PTP1B in these cells. Insulin-induced IR and IRS1 tyrosyl phosphorylation was elevated in cells with abolished/minimal PTP1B activity (KO and D/A, respectively) and attenuated in those with increased/prolonged PTP1B activity (K/R). Similarly, PPARγ, C/EBPα, and PGC1α mRNA was increased in WT, K/O and D/A cells compared with K/R. Notably, our studies demonstrated regulation of insulin-stimulated glucose uptake in adipocytes by PTP1B, but that did not fully correlate with alterations in IR and IRS1 tyrosyl phosphorylation and Glut4 expression. This in line with previous studies [52], [53], [54], [55] that suggest that a multitude of factors are required to evoke maximal insulin-stimulated glucose transport including, but not limited to, modulation of signaling at specific intracellular compartments. Collectively, our findings indicate that alteration of insulin signaling via modulation of PTP1B activity accounts, at least in part, for the observed differentiation effects.

PTP1B also can regulate brown fat adipogenesis through insulin-independent signaling pathways (Fig. 8). AMPK has been implicated in the regulation of brown fat adipogenesis. Inhibition of AMPK blocks brown but not white adipocyte differentiation, and chronic activation of AMPK *in vivo* increases brown adipocytes within WAT depots [47]. Notably, AMPK activity is elevated, and AMPK target genes that regulate mitochondrial biogenesis are induced in BAT of PTP1B KO mice [34]. In line with this, our data demonstrated regulation of AMPK signaling by PTP1B in brown adipocytes. Collectively, these findings indicate that PTP1B can regulate brown adipose differentiation, at least in part, through AMPK signaling. Finally, we cannot rule out modulation of additional affectors(s) of brown adipose differentiation by PTP1B (Fig. 8). Adipogenic differentiation is associated with downregulation of Wnt/β-catenin signaling [56], [57], [58]. Since PTP1B has been implicated in regulating β-catenin signaling [59], [60] it is tempting to speculate that it could influence adipogenesis through this signaling pathway. Proper execution of adipogenesis requires integration of a wide array of stimuli and regulated expression of numerous genes, so it is not surprising that PTP1B participates in this complex process through regulating various signaling pathways.

In summary, our studies identify PTP1B as a modulator of brown fat adipogenesis, and suggest that adipocyte differentiation requires regulated expression of PTP1B. These findings are of direct relevance to obesity and diabetes given the contribution of brown fat to energy homeostasis, and considering that PTP1B is a target that is being harnessed as a potential therapeutic.

## Materials and Methods

### Chemicals and reagents

Dulbecco's Modified Eagle Medium (DMEM), fetal bovine serum (FBS) and trypsin were purchased from Invitrogen (Carlsbad, CA). Antibodies for human PTP1B (FG6), mouse PTP1B, IR, IRS1 were purchased from Upstate Biotechnology (Lake Placid, NY), pAkt (Ser473), pErk, pAMPK (Thr172), AMPK, pACC (Ser79), ACC were purchased from Cell Signaling Technology (Beverly, MA), LKB1, Tubulin, Akt and Erk were from Santa Cruz Biotechnology (Santa Cruz, CA). Horseradish peroxidase (HRP)-conjugated secondary antibodies were purchased from BioResources International (Carlsbad, CA). Unless otherwise indicated, chemicals were purchased from Sigma (St. Louis, MO).

### Cell isolation and culture

Brown adipocytes and their precursor cells were isolated from newborn wild type and whole-body PTP1B KO mice by collagenase digestion as described previously [61]. Preadipocytes were immortalized by infection with the retroviral vector pBABE encoding SV40T-antigen then selected with puromycin (2 µg/ml). To induce cell differentiation, preadipocytes were grown to confluence in culture medium containing 20% FBS. Confluent cells were then switched to differentiation media containing 20% FBS, 20 nM insulin and 1 nM triiodothyronine [T3] for 48 hours. Adipocyte differentiation was induced by treating cells for 48 h in differentiation medium further supplemented with 0.5 µM dexamethasone, 0.5 mM isobutylmethylxanthine, and 0.125 mM indomethacin (induction media). After induction, cells were returned to differentiation medium, and at day 8 exhibited a fully differentiated phenotype with massive accumulation of multilocular fat droplets. All animal work was conducted following federal guidelines and in accordance with University of California Davis IACUC approval (protocol # 13064).

For oil red O staining, cells were fixed with 10% buffered formalin for at least 1 hour at room temperature. Cells were then stained for one hour with filtered oil red O solution (5g/liter in isopropyl alcohol), washed with distilled water, and visualized. Oil red O was quantified spectrophotometrically at 520 nm.

PTP1B KO cell lines were reconstituted using retrovirus encoding human PTP1B wild type (WT), substrate-trapping mutant PTP1B D/A [37], and sumoylation-resistant mutant PTP1B K/R [38] as we previously described [36]. Briefly, viral Φ NX-packaging cells were transfected with retroviral vectors using Lipofectamine 2000 (Invitrogen) following manufacturer's instructions and viral supernatants were harvested 48 h after transfection. PTP1B KO cells were infected with polybrene (4 µg/ml)-supplemented virus-containing supernatants. Selection was started 48 hours after infection with 200 µg/ml of hygromycin (Invitrogen) and pools of drug-resistant cells maintained.

### Biochemical analyses

For signaling experiments, cells were starved overnight then stimulated with insulin (100 nM) for 5 or 10 minutes. Cells were lysed using radio-immunoprecipitation assay (RIPA) buffer (10 mM Tris-HCl, pH 7.4, 150 mM NaCl, 0.1% sodium dodecyl sulfate [SDS], 1% Triton X-100, 1% sodium deoxycholate, 5 mM EDTA, 1 mM NaF, 1 mM sodium orthovanadate and protease inhibitors). Extracts were sonicated and clarified by centrifugation at 13,000 rpm for 10 min, and protein concentrations were determined using a bicinchoninic acid protein assay kit (Pierce Chemical, Rockford, IL). Proteins (500–1000 µg) were subjected to immunoprecipitation using IR, IRS1 and phosphotyrosine (4G10) antibodies. For substrate-trapping experiments, lysates were prepared in 1% NP40 buffer with a protease inhibitor cocktail (without sodium orthovanadate) and hPTP1B was immunoprecipitated using FG6 antibodies. Immune complexes were collected on protein G-Sepharose beads (GE Healthcare) and washed with lysis buffer. Proteins were resolved by SDS-PAGE and transferred to PVDF membranes. Immunoblotting of total cell lysates and immunoprecipitates was performed with antibodies for phosphotyrosine (1/10,000), IR (1/1,000), IRS1 (1/500), pErk (Thr202/Tyr204) (1/20,000), pAkt (Ser473) (1/10,000), Erk (1/10,000), Akt (1/5,000), UCP1 (1/5,000), pAMPKα (1/2,000), AMPKα (1/2,000), pACC (1/1,000), LKB1 (1/2,000) and Tubulin (1/1,000). After incubation with appropriate secondary antibodies, proteins were visualized using enhanced chemiluminescence (Amersham Biosciences). Pixel intensities of immunoreactive bands were quantified using FluorChem 9900 (Alpha Innotech).

RNA was extracted from differentiated adicpocytes using TRIzol reagent (Invitrogen). cDNA was generated using high-capacity cDNA Archive Kit (Applied Biosystems). Expression of PPARγ, C/EBPα, C/EBPδ, PGC1α and Pref1 was assessed by quantitative real-time PCR (iCycler, BioRad) with appropriate primers (Table S1) and normalized to glyceraldehyde 3-phosphate dehydrogenase (GAPDH).

### Glucose uptake assay

Cells were assayed for glucose uptake essentially as described [62]. Briefly, differentiated brown adipocytes were treated with insulin for 30 minutes after which 2-deoxy-[^3^H] glucose (0.5 µCi/ml, final concentration) was added for an additional 3 minutes. The incorporated radioactivity was quantitated using liquid scintillation counting.

### Statistical analyses

Data are expressed as means ± standard error of the mean (SEM). Statistical analyses were performed using JMP program (SAS Institute). Comparisons between groups were made by unpaired two-tailed Student's *t* test. Differences were considered significant at P≤0.05 and highly significant at P≤0.01. A single symbol (such as *) indicates P≤0.05 and a double symbol (**) indicates P≤0.01.

## Supporting Information

Table S1 **Primers used for real time PCR.** Primer sequences used to determine mRNA expression levels of PPARγ, C/EBPα, C/EBPδ, Pref1, PGC1α and GAPDH in brown adipocytes during differentiation.
(DOC)
