# Expression of Concern: Regulation of Brown Fat Adipogenesis by Protein Tyrosine Phosphatase 1B

**DOI:** 10.1371/journal.pone.0296401

**Published:** 2023-12-21

**Authors:** The PLOS ONE Editors

Following the publication of this article [**1**], concerns were raised regarding the results presented in Figs 1 and 5. Specifically,

- In Fig 1A, the following bands appear similar:
  ○ The band representing K/R in the mPTP1B panel and the band representing Con in the Tubulin panel
  ○ The bands representing KO and D/A in the Tubulin panel

The Fig 5A Tubulin panel of this article [**1**] appears similar to lanes 2–13 of the Fig 3C STAT3 panel of [2] when rotated 180°. The corresponding author commented that he is not aware of rearranging, splicing, or cutting of blots during figure preparation and that the original data underlying Figs 1 and 5 are no longer available. Instead, the corresponding author provided supporting data from repeat experiments, which are available in the S1, S8, and S9 Files provided with this notice. In the absence of the original underlying data, the concerns with Figs 1A and 5A cannot be fully resolved and these results should be interpreted with caution.

In following up on these issues, the authors provided the available underlying data for other figures in the article (S2–S7 Files).

The *PLOS ONE* Editors issue this Expression of Concern to notify readers of the above concerns and to relay the available data provided by the corresponding author.

The Fig. 5A Tubulin panel reports material from [2], published in 2011 by Bettaieb et al, under a CC-BY 4.0 DEED license. At the time of publication of this Expression of Concern, this article [1] was republished to update the Fig 5 legend to provide correct attribution.

## Supporting information

S1 File Repeat Experiment data provided in support of the Fig 1A results. (PDF)

S2 File Image data underlying the Fig 1C results. (PDF)

S3 File Original image data underlying Fig 3A results. (PDF)

S4 File Original image data underlying Fig 3B and 3C results. (PDF)

S5 File Original image data underlying Fig 3C results. (PDF)

S6 File Original image data underlying Fig 4D, 4E, 4F, and 4G results. (PDF)

S7 File Original image data underlying Fig 4G results. (PDF)

S8 File Repeat Experiment data provided in support of the Fig 5A results. (PDF)

S9 File Repeat Experiment data provided in support of the Fig 5C results. (PDF)
