# Supplementary material for: Regulation of Brown Fat Adipogenesis by Protein Tyrosine Phosphatase 1B
Source: PLoS One. 2011 Jan 31;6(1):e16446. doi: 10.1371/journal.pone.0016446 (PMC3031545; doi:10.1371/journal.pone.0016446)
Supplement: Table S1 — Primers used for real time PCR. Primer sequences used to determine mRNA expression levels of PPARγ, C/EBPα, C/EBPδ, Pref1, PGC1α and GAPDH in brown adipocytes during differentiation. (DOC) [file pone.0016446.s001.doc]

| **Gene** | **Sequence (5′→3′)** | |
| --- | --- | --- |
| PPARγ | Forward | TCGCTGATGCACTGCCTATG |
|  | Reverse | GAGAGGTCCACAGAGCTGATT |
| C/EBPα | Forward | CAAGAACAGCAACGAGTACCG |
|  | Reverse | GTCACTGGTCAACTCCAGCAC |
| C/EBPδ | Forward | CGACTTCAGCGCCTACATTGA |
|  | Reverse | CTAGCGACAGACCCCACAC |
| Pref1 | Forward | AGTACGAATGCTCCTGCACAC |
|  | Reverse | CTGGCCCTCATCATCCAC |
| PGC1α | Forward | TATGGAGTGACATAGAGTGTGCT |
|  | Reverse | CCACTTCAATCCACCCAGAAAG |
| GAPDH | Forward | ACTGAGGACCACCAGGTTGTC |
|  | Reverse | TGCTGTAGCCGTATTCATTG |
