# Supplementary figures and images for: Expression of Concern: Regulation of Brown Fat Adipogenesis by Protein Tyrosine Phosphatase 1B
Source: PLoS One. 2023 Dec 21;18(12):e0296401. doi: 10.1371/journal.pone.0296401 (PMC10735039; doi:10.1371/journal.pone.0296401)

**A**

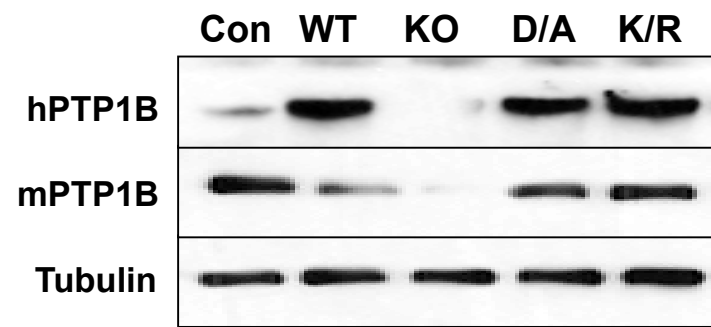

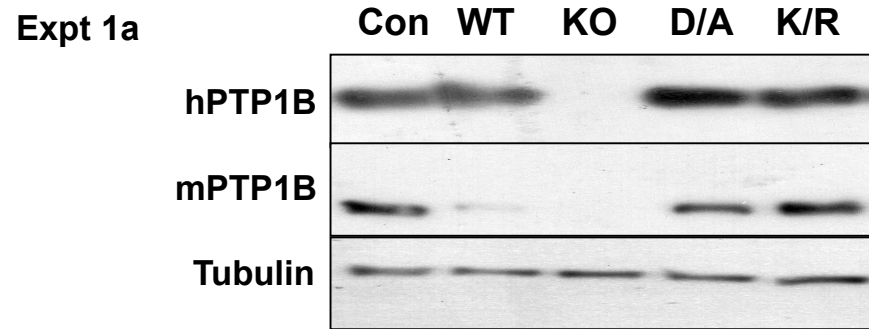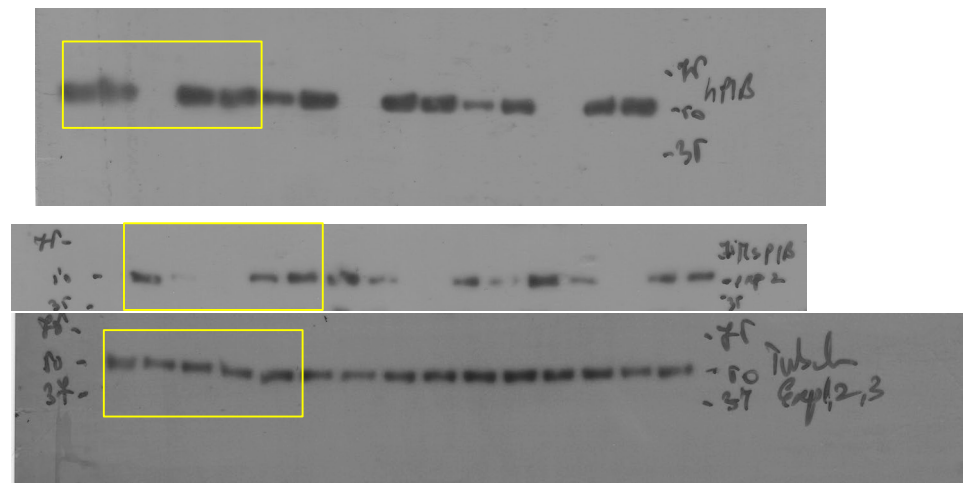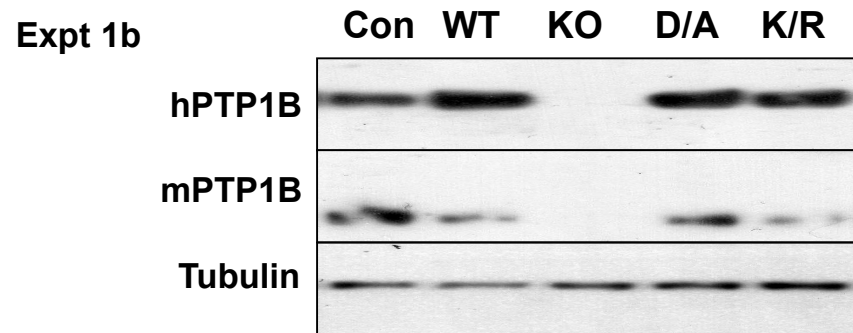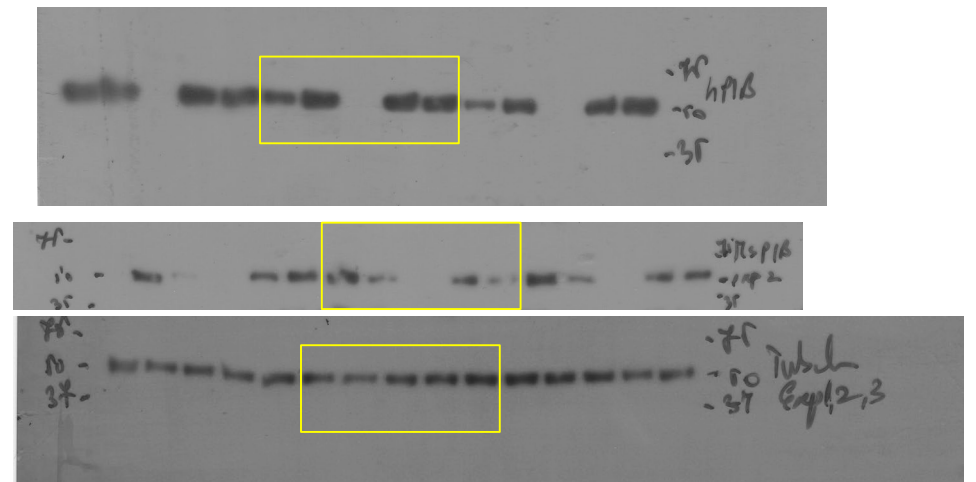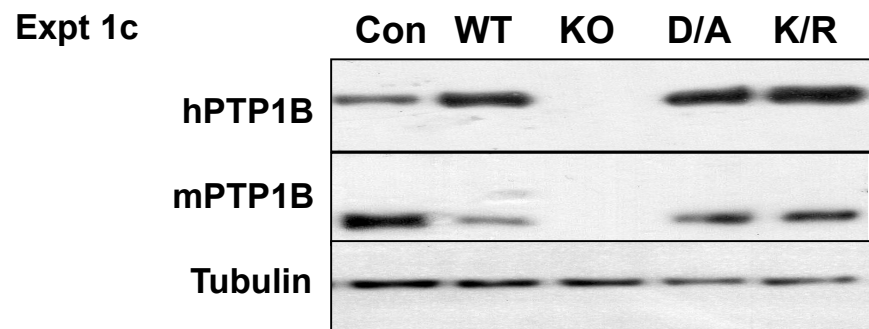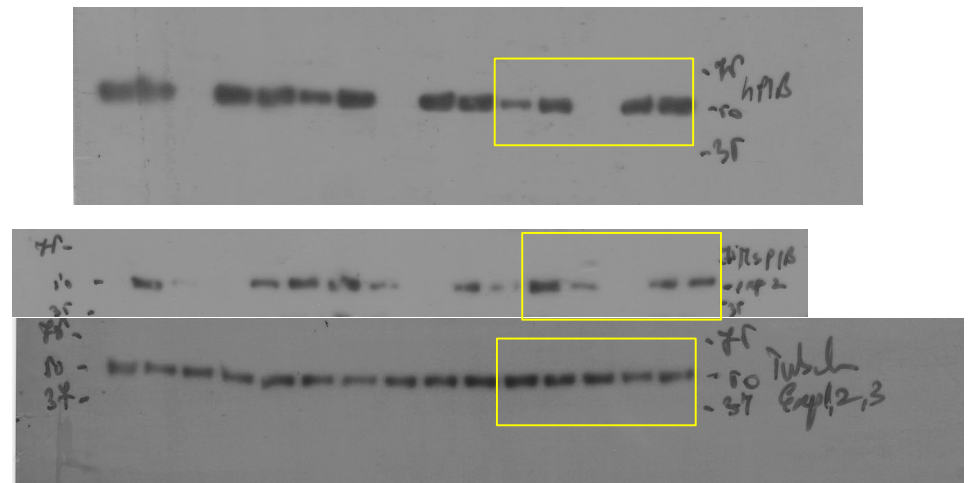

Expt 2a

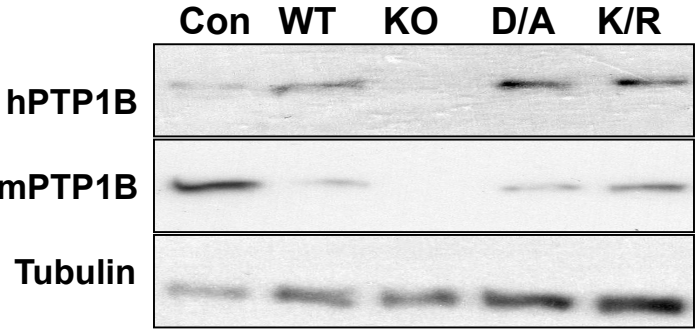

Expt 2b

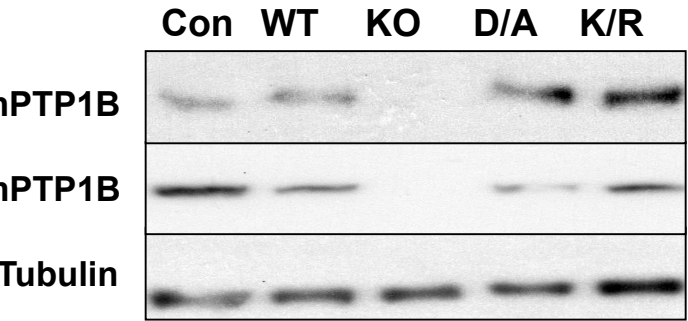

Expt 2c

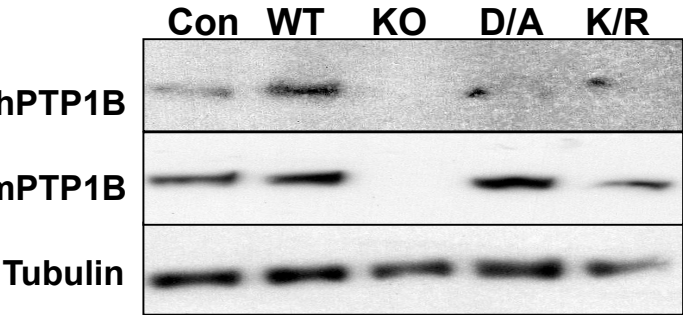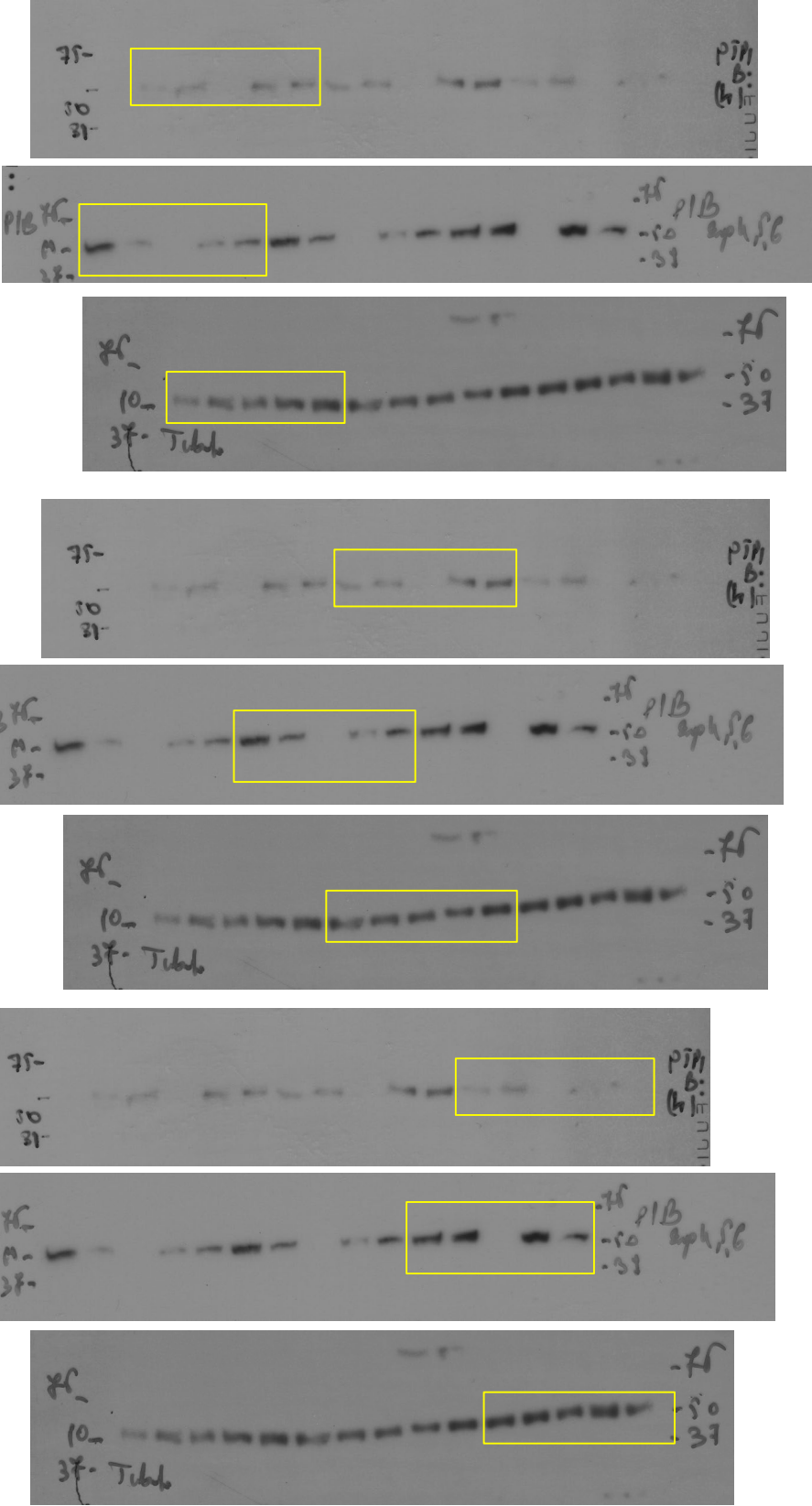

Supportive data for Matsuo and Bettaieb *et al.*, Fig. 1A

Supplement: S1 File — (PDF) [file pone.0296401.s001.pdf]

**A**

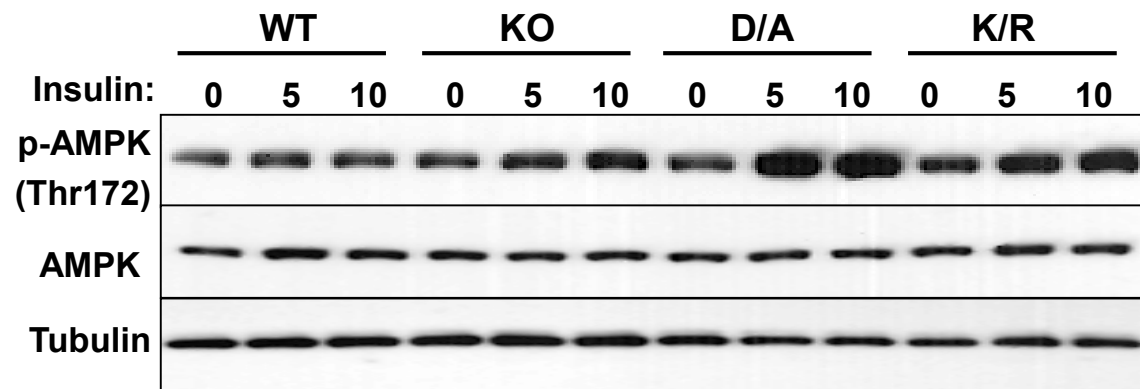

Expt 1

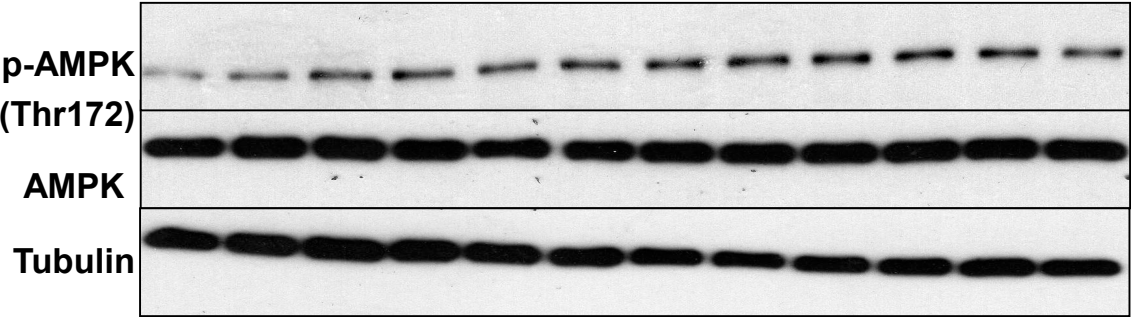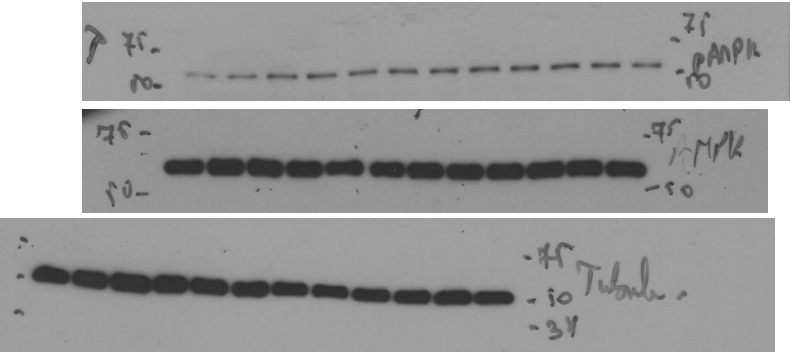

Expt 2

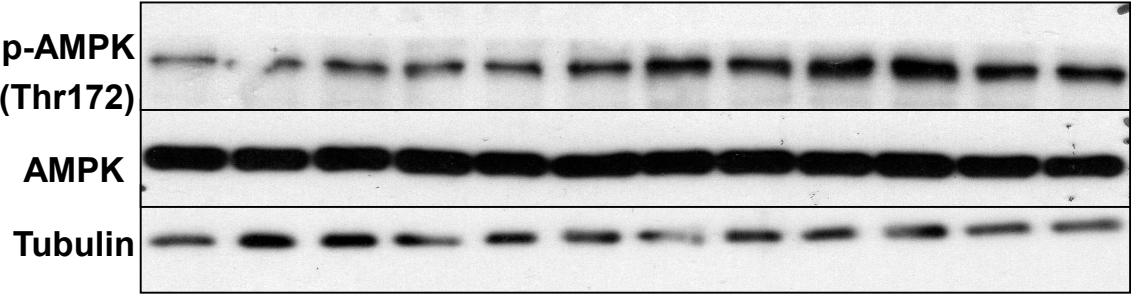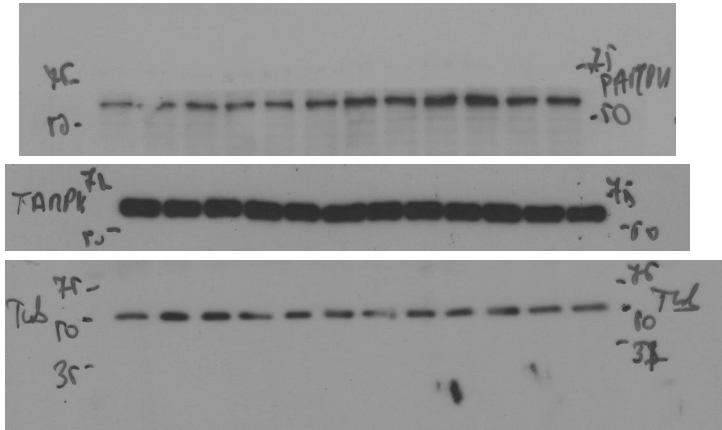

Expt 3

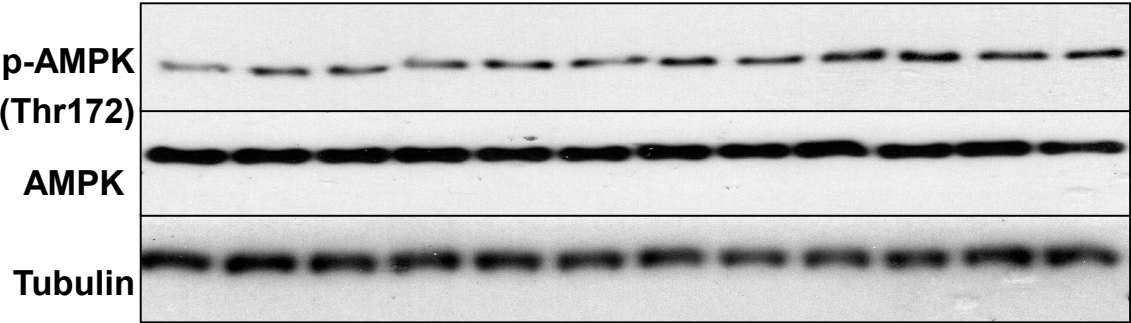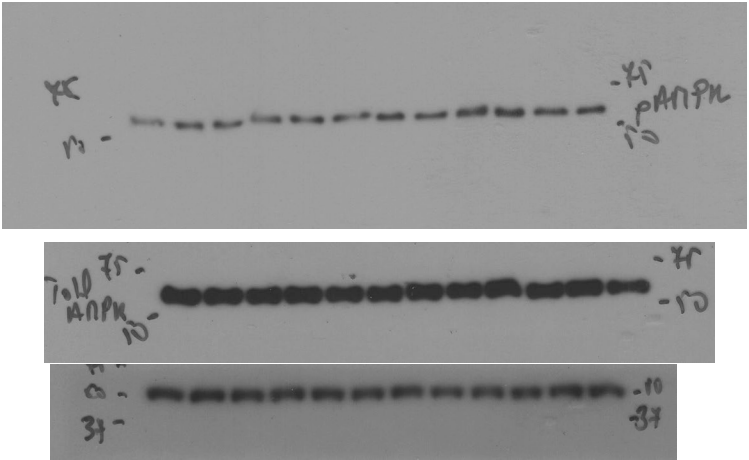

Supplement: S8 File — (PDF) [file pone.0296401.s008.pdf]

C

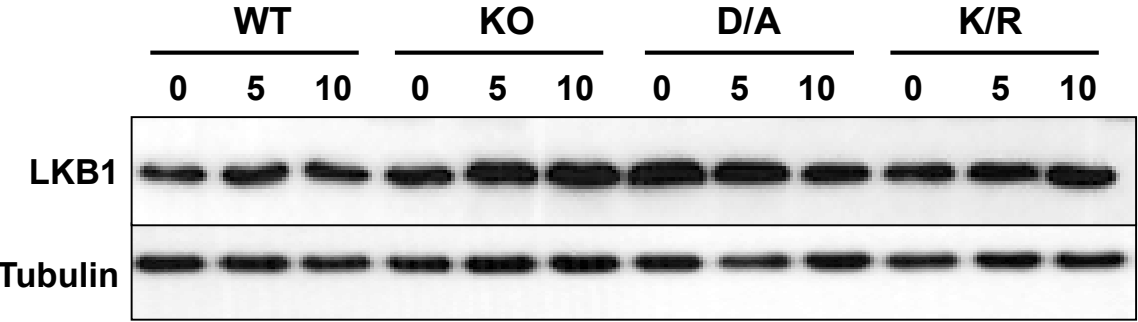

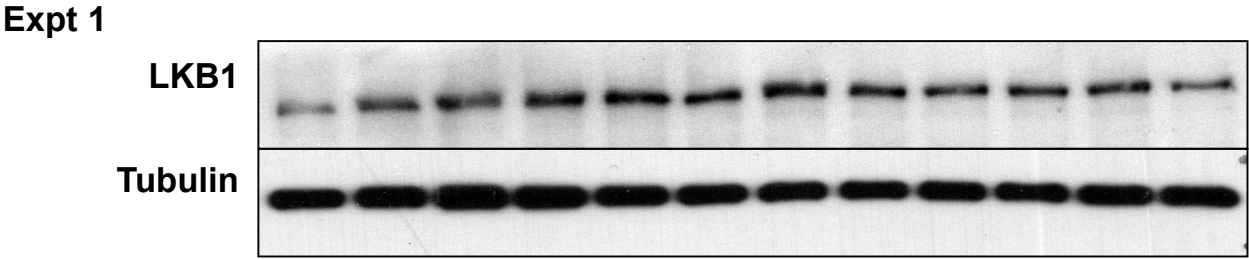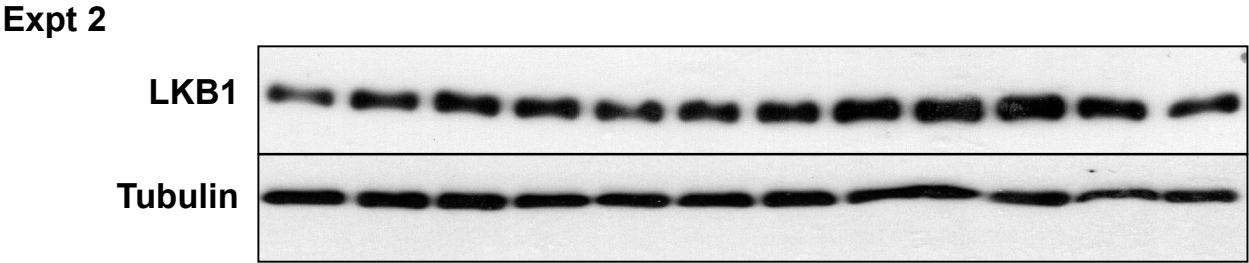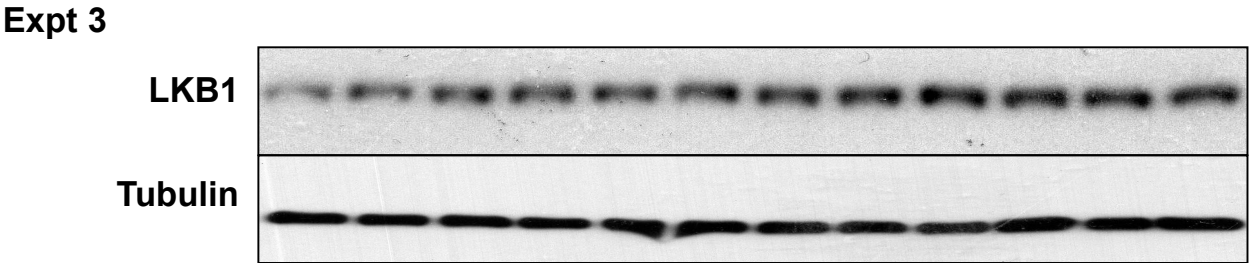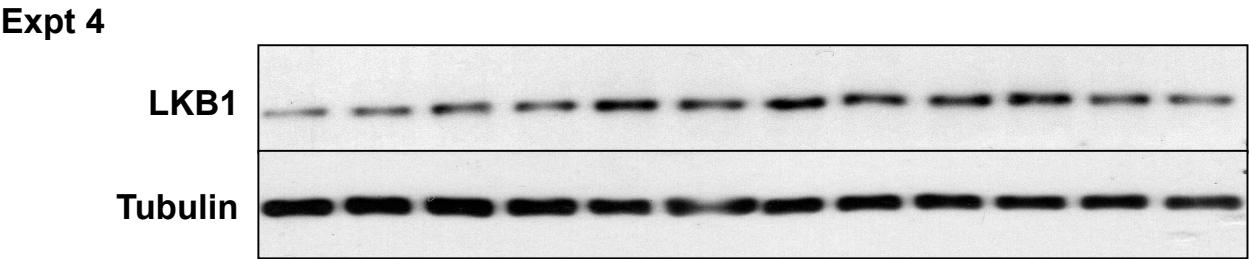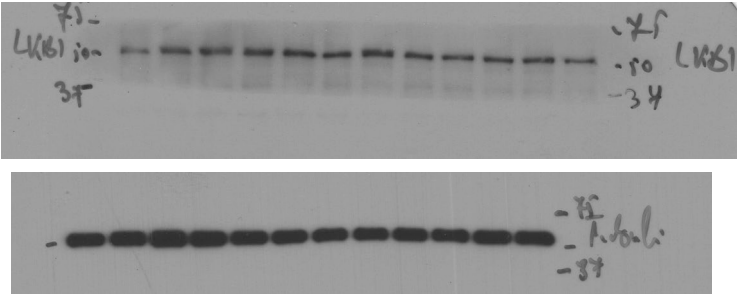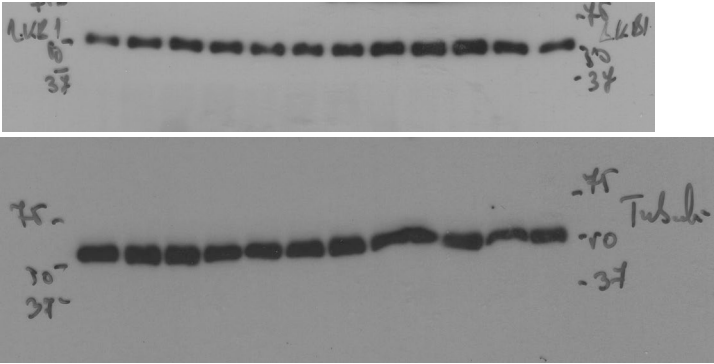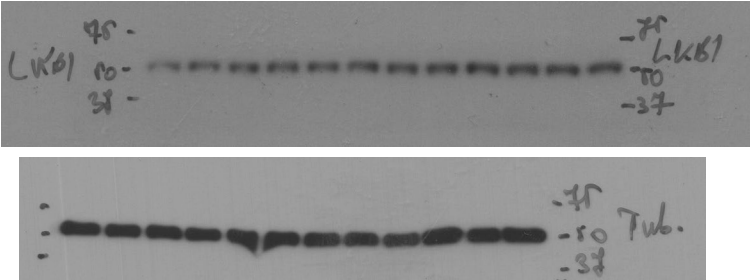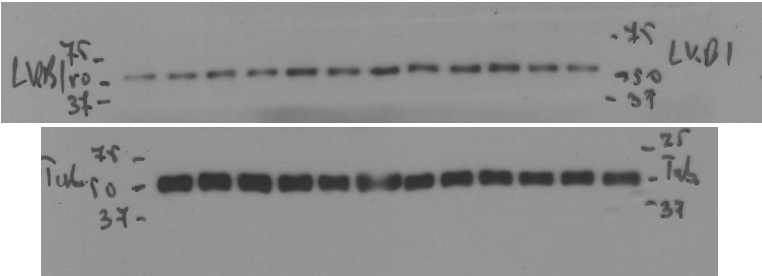

Supportive data for Matsuo and Bettaieb *etal.*, Fig. 5C

Supplement: S9 File — (PDF) [file pone.0296401.s009.pdf]
