# Supplementary material for: Expression of Concern: Regulation of Brown Fat Adipogenesis by Protein Tyrosine Phosphatase 1B
Source: PLoS One. 2023 Dec 21;18(12):e0296401. doi: 10.1371/journal.pone.0296401 (PMC10735039; doi:10.1371/journal.pone.0296401)

Different experimental groups of oil red O staining (Supporting the data in Figure 1)

Fat cell differentiation and oil red O staining (group 1)

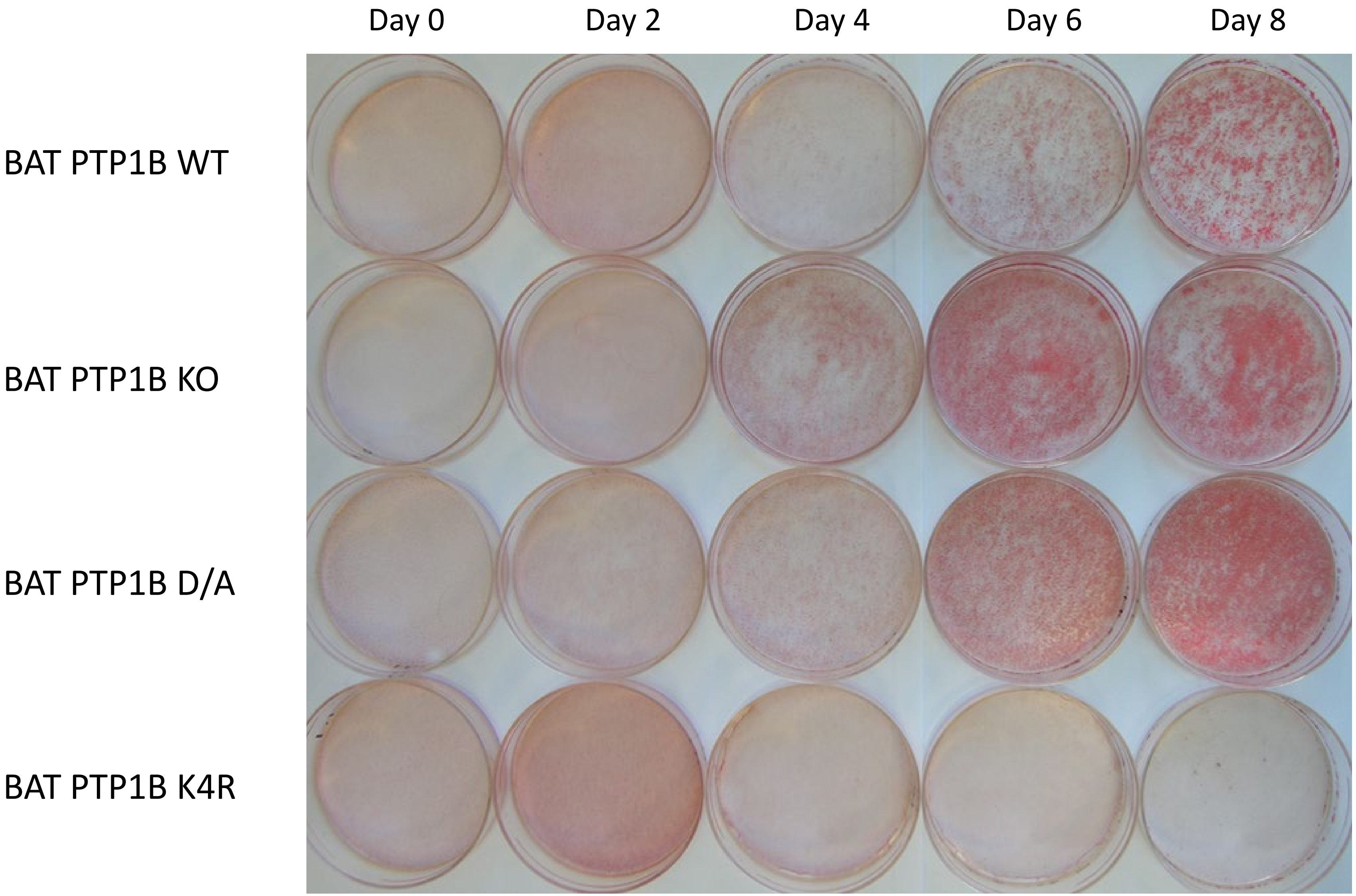

Fat cell differentiation and oil red O staining (group 2)

Day 0 | Day 2 | Day 4 | Day 6 | Day 8 | Day 10

BAT PTP1B WT

BAT PTP1B KO

BAT PTP1B D/A

BAT PTP1B K4R

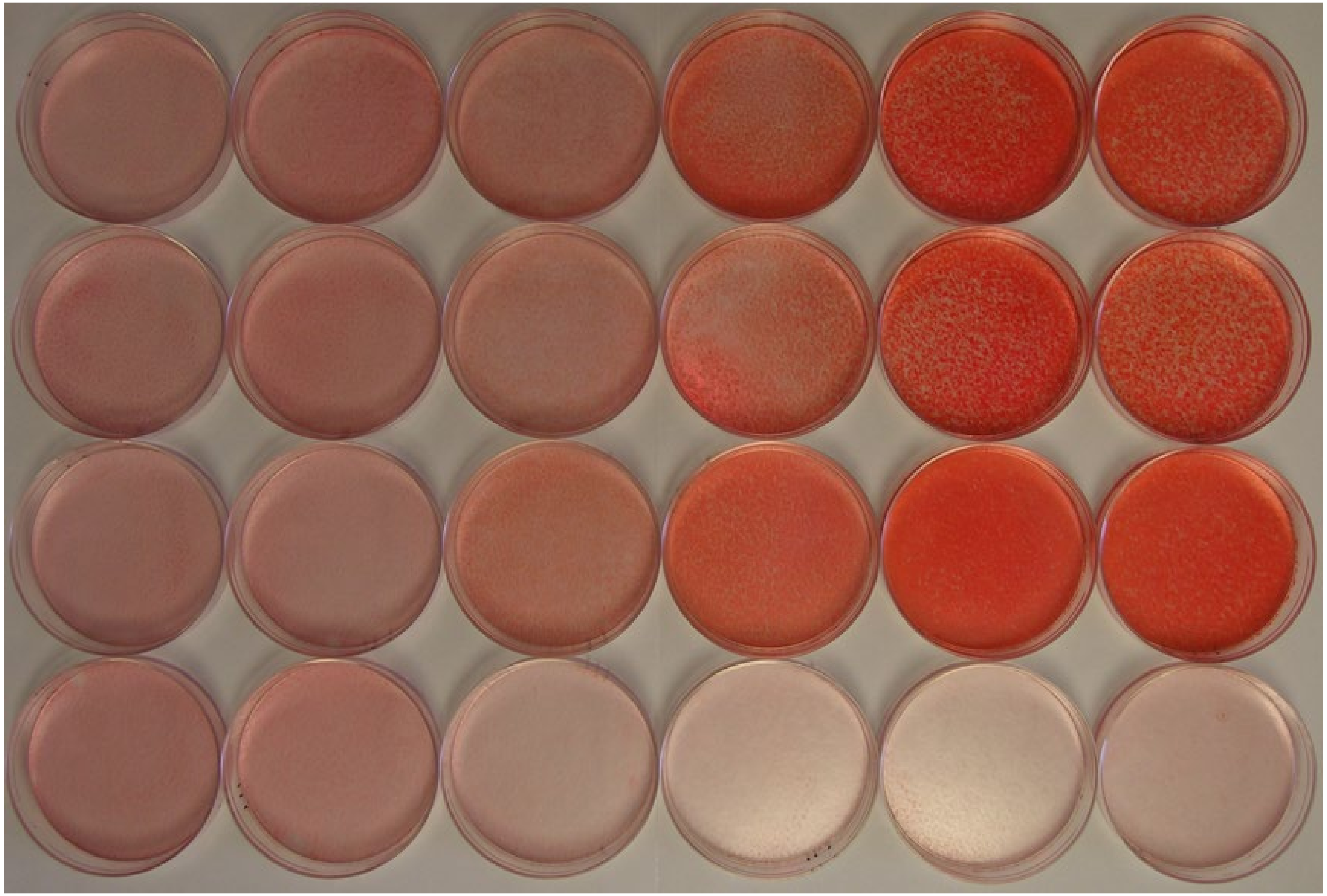

Fat cell differentiation and oil red O staining (group 3)

Day 0 Day 2 Day 4 Day 6 Day 8

BAT PTP1B WT

BAT PTP1B KO

BAT PTP1B D/A

BAT PTP1B K4R

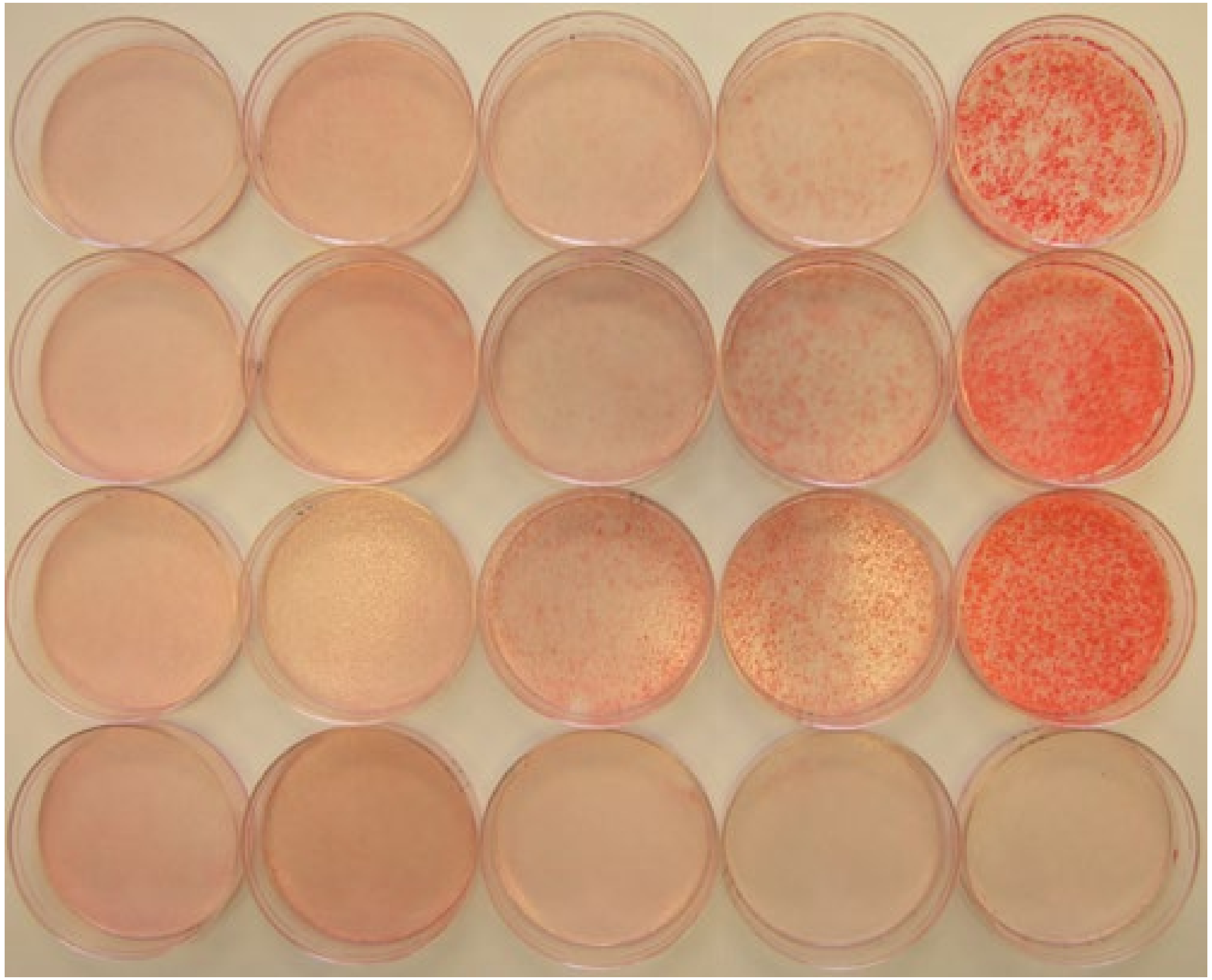

Supplement: S2 File — (PDF) [file pone.0296401.s002.pdf]
