# Supplementary material for: Expression of Concern: Regulation of Brown Fat Adipogenesis by Protein Tyrosine Phosphatase 1B
Source: PLoS One. 2023 Dec 21;18(12):e0296401. doi: 10.1371/journal.pone.0296401 (PMC10735039; doi:10.1371/journal.pone.0296401)
Supplement: S3 File — (PDF) [file pone.0296401.s003.pdf]

Scans of the original p-Tyr and PTP1B blots in Fig. 3A

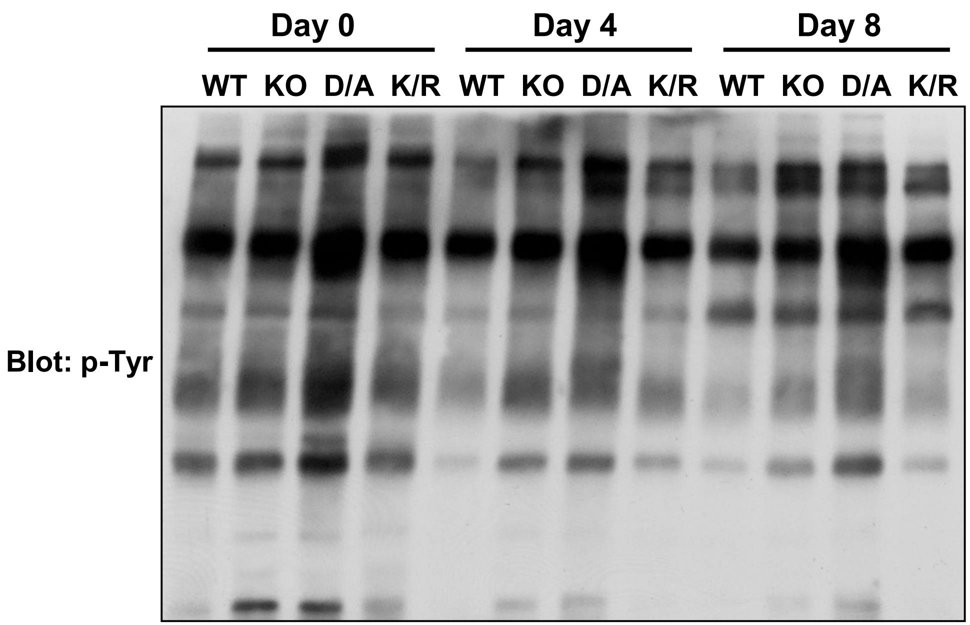

Fig. 3A, p-Tyr

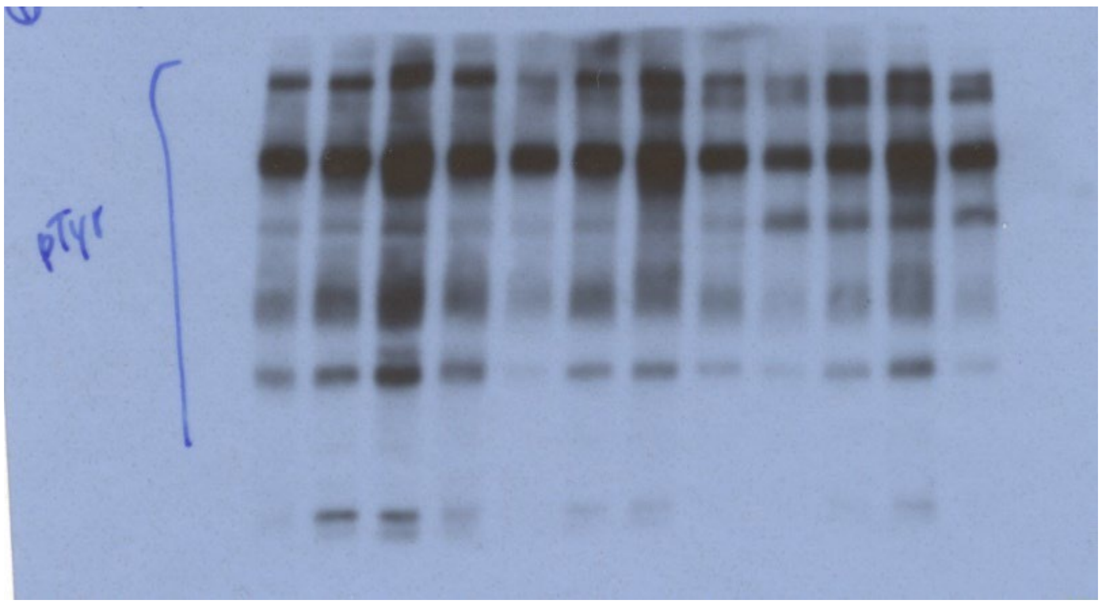

Scan of the original blot

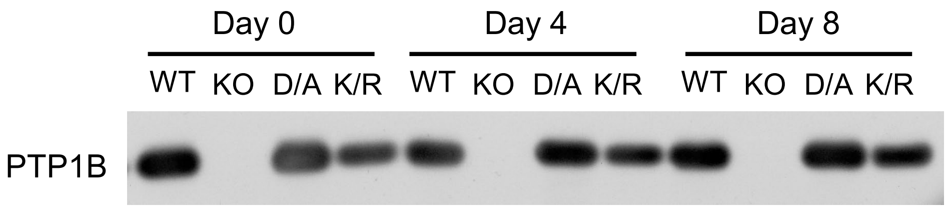

Fig. 3A, PTP1B

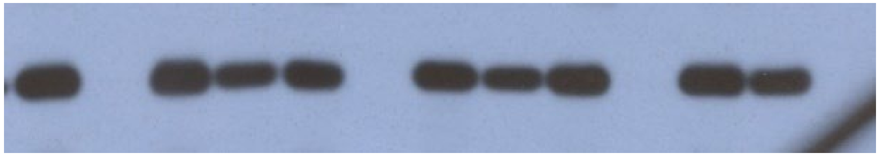

Scan of the original blot
