# Supplementary material for: Expression of Concern: Regulation of Brown Fat Adipogenesis by Protein Tyrosine Phosphatase 1B
Source: PLoS One. 2023 Dec 21;18(12):e0296401. doi: 10.1371/journal.pone.0296401 (PMC10735039; doi:10.1371/journal.pone.0296401)

Scans of original blots for p-Erk/Erk and p-Akt/Akt in figures 3B and 3C, respectively

B

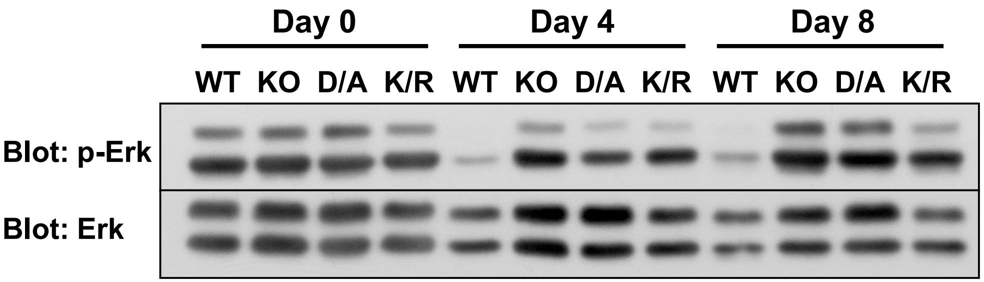

C

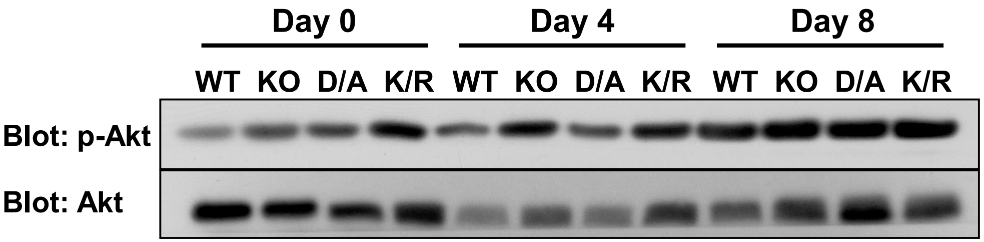

p-Erk

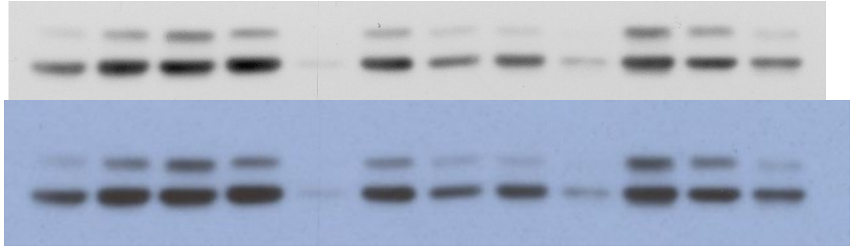

p-Akt

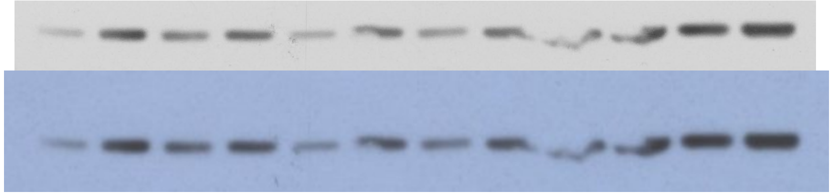

p-Erk

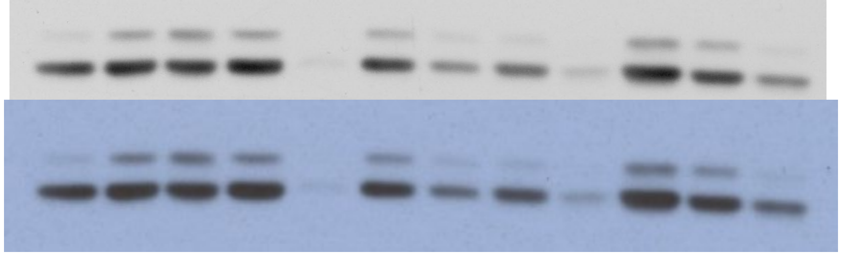

p-Akt

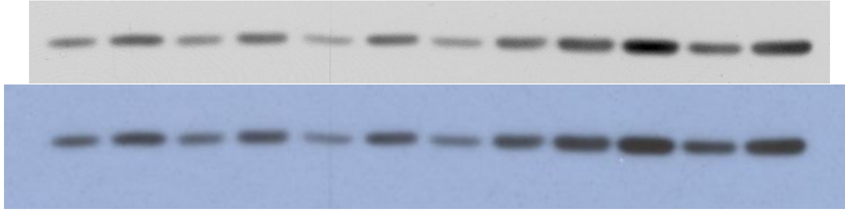

Erk

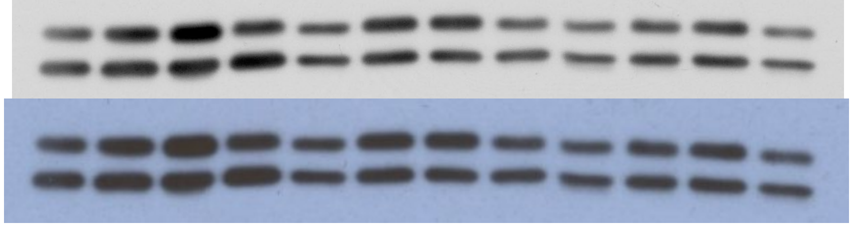

Akt

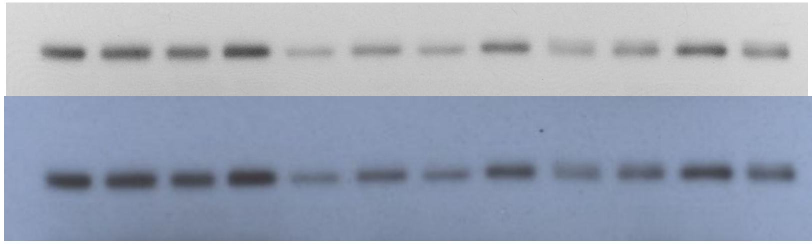

Supplement: S4 File — (PDF) [file pone.0296401.s004.pdf]
