# Supplementary material for: Expression of Concern: Regulation of Brown Fat Adipogenesis by Protein Tyrosine Phosphatase 1B
Source: PLoS One. 2023 Dec 21;18(12):e0296401. doi: 10.1371/journal.pone.0296401 (PMC10735039; doi:10.1371/journal.pone.0296401)

Supportive individual data on quantitating IR phosphorylation (Fig. 4D)

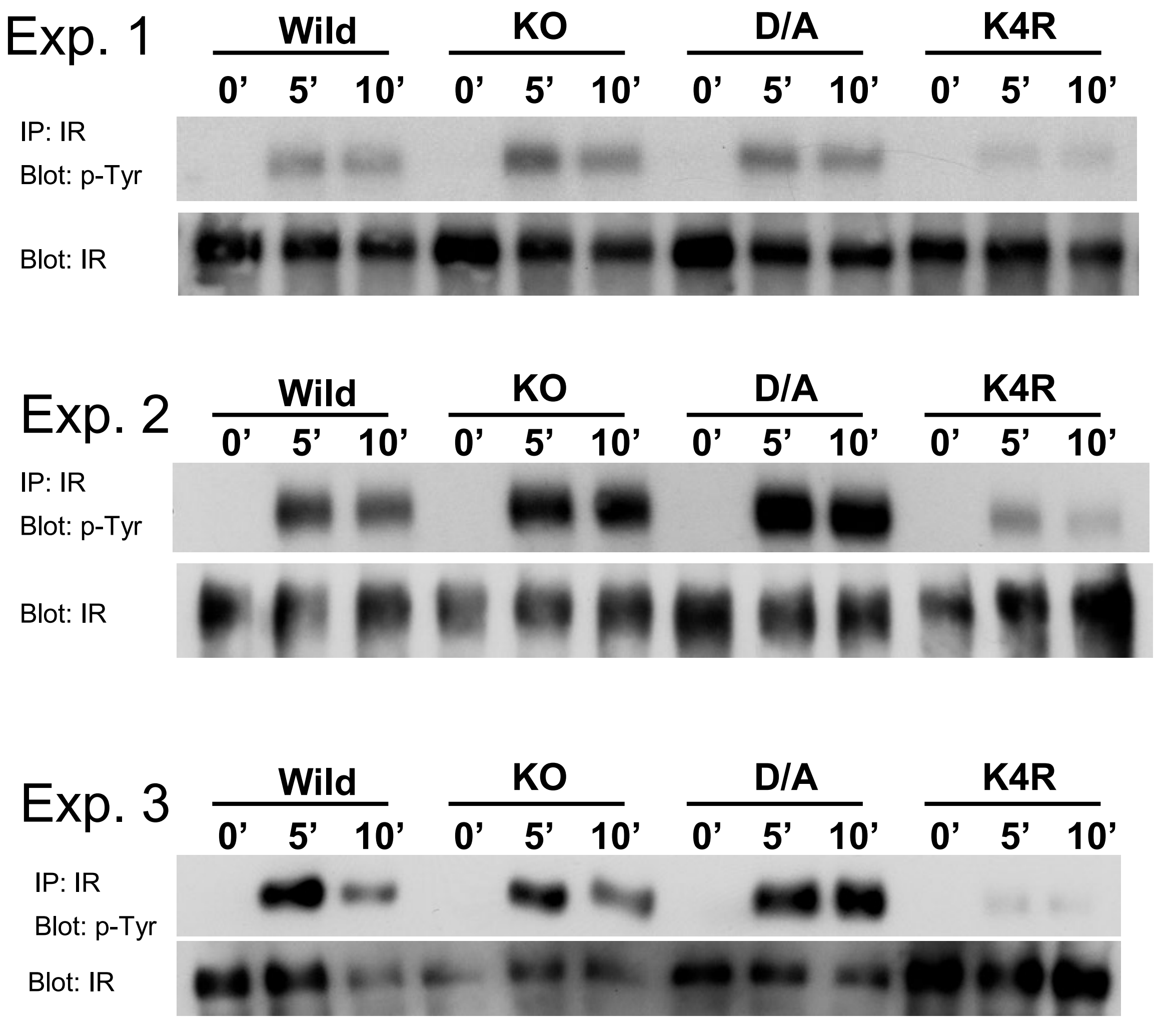

Supportive individual data on quantitating IRS-1 phosphorylation (Fig. 4E)

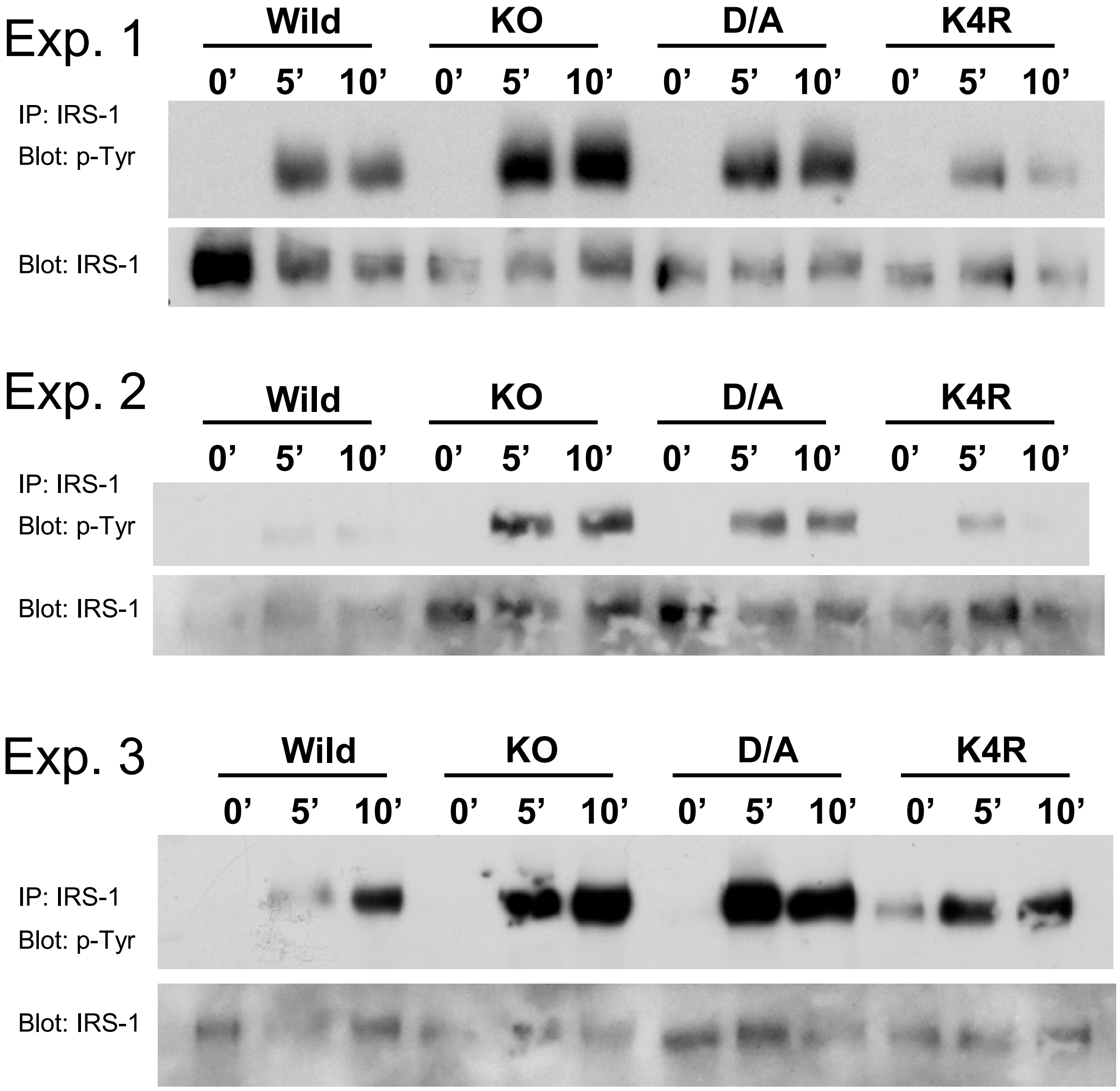

Supportive individual data on quantitating Erk phosphorylation (Fig. 4F)

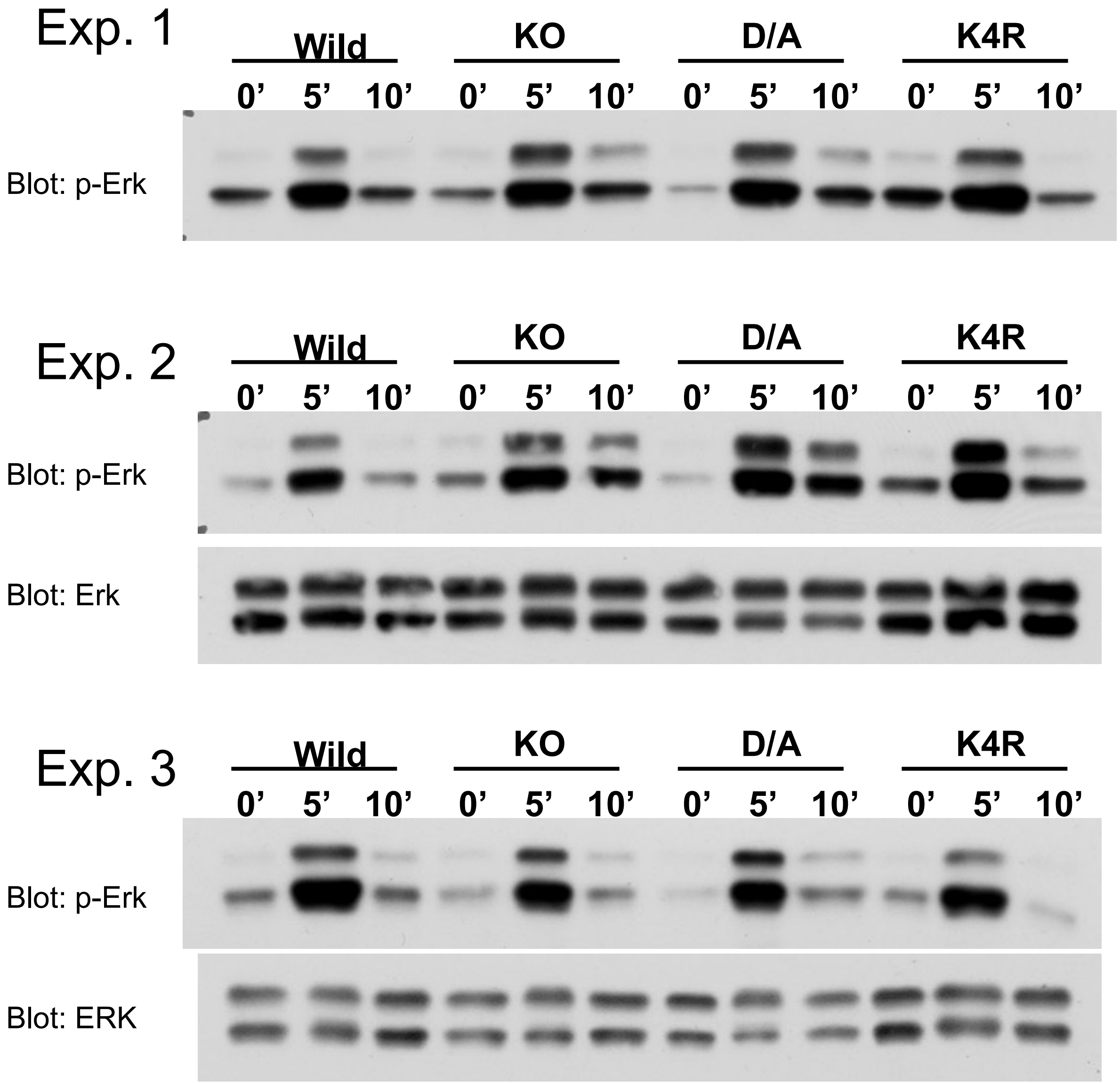

Supportive individual data on quantitating Akt phosphorylation (Fig. 4G)

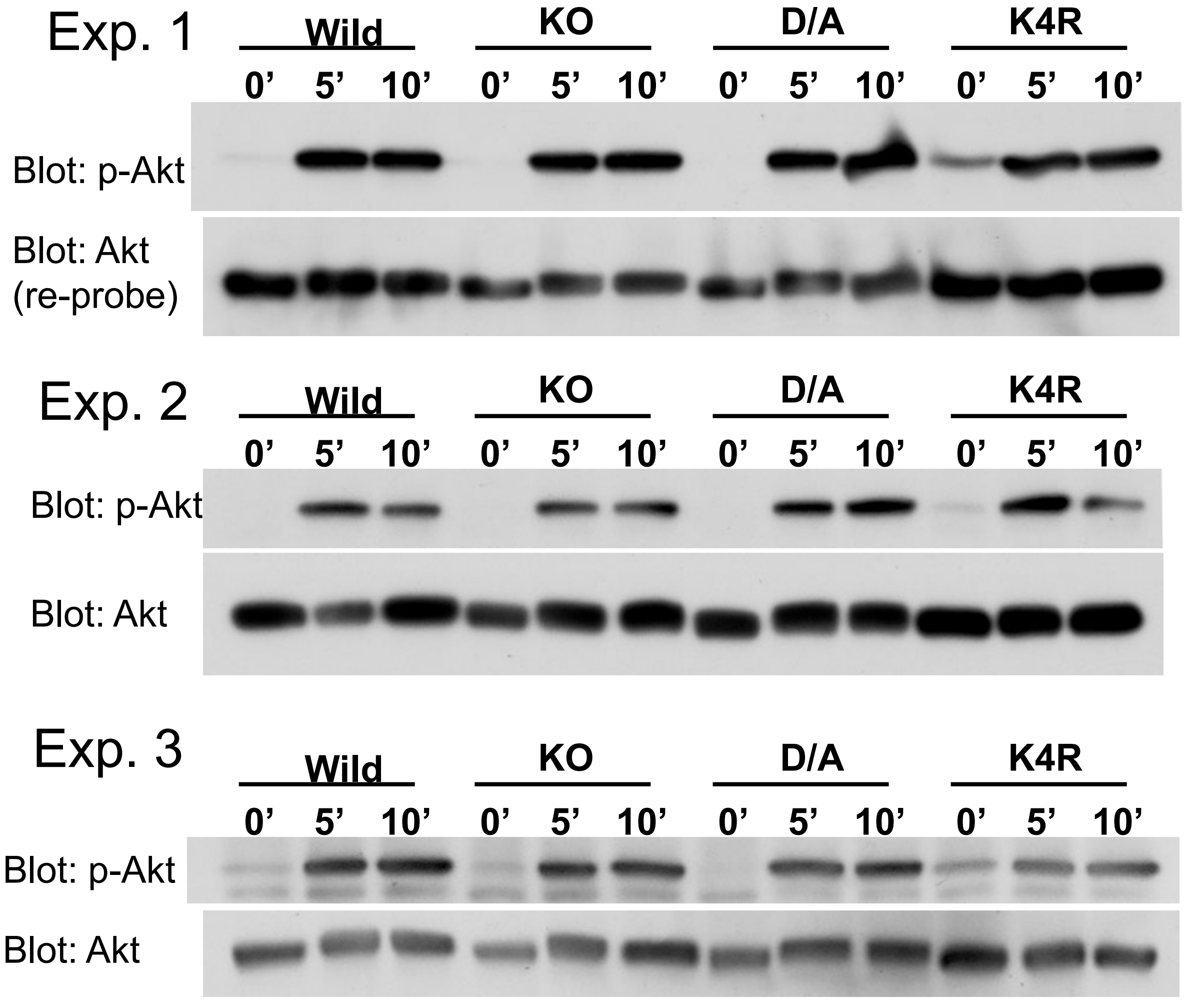

Supplement: S6 File — (PDF) [file pone.0296401.s006.pdf]
