# Supplementary material for: Expression of Concern: Regulation of Brown Fat Adipogenesis by Protein Tyrosine Phosphatase 1B
Source: PLoS One. 2023 Dec 21;18(12):e0296401. doi: 10.1371/journal.pone.0296401 (PMC10735039; doi:10.1371/journal.pone.0296401)
Supplement: S7 File — (PDF) [file pone.0296401.s007.pdf]

Scans of the original p-Akt and Akt blots in Fig. 4G

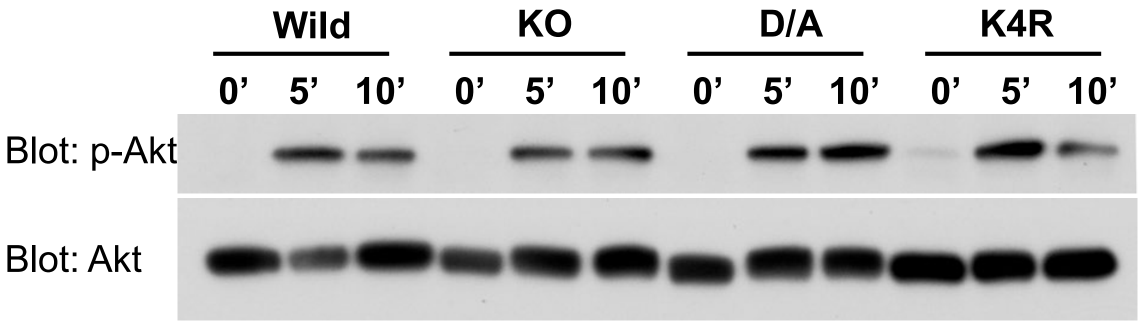

Fig. 4G, p-Akt and Akt

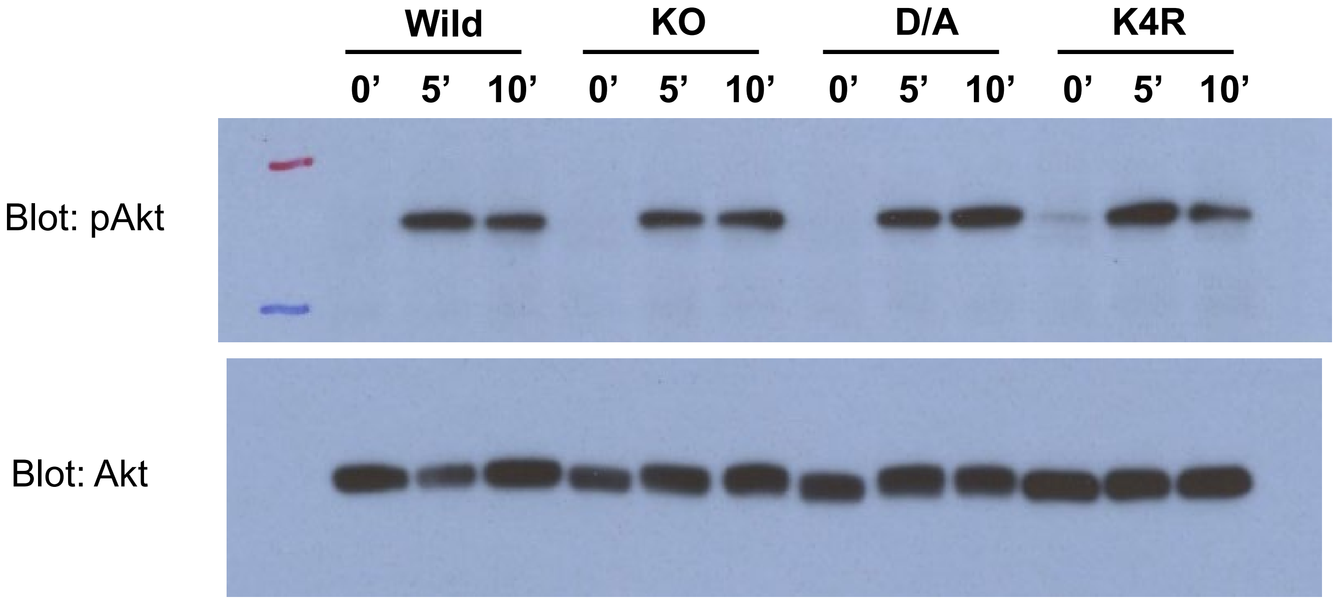

Scan of the original p-Akt blot (markings on the left indicate markers of MW)

Scan of the original Akt blot
